# Mitosis Is a Source of Potential Markers for Screening and Survival and Therapeutic Targets in Cervical Cancer

**DOI:** 10.1371/journal.pone.0055975

**Published:** 2013-02-06

**Authors:** Ana María Espinosa, Ana Alfaro, Edgar Roman-Basaure, Mariano Guardado-Estrada, Ícela Palma, Cyntia Serralde, Ingrid Medina, Eligia Juárez, Miriam Bermúdez, Edna Márquez, Manuel Borges-Ibáñez, Sergio Muñoz-Cortez, Avissai Alcántara-Vázquez, Patricia Alonso, José Curiel-Valdez, Susana Kofman, Nicolas Villegas, Jaime Berumen

**Affiliations:** 1 Departamento de Medicina Experimental, Facultad de Medicina, Universidad Nacional Autónoma de México, México City, Mexico; 2 Unidad de Medicina Genómica, Hospital General de México, México City, Mexico; 3 Servicio de Oncología, Hospital General de México, México City, Mexico; 4 Escuela Superior de Medicina, Instituto Politécnico Nacional, México City, Mexico; 5 Instituto de Investigación en Matemáticas Aplicadas, Universidad Nacional Autónoma de México, México City, Mexico; 6 Servicio de Ginecobstetricia, Hospital General de México, México City, Mexico; 7 Servicio de Patología, Hospital General de México, México City, Mexico; 8 Servicio de Genética, Hospital General de México, México City, Mexico; 9 Departamento de Biomedicina Molecular, Centro de Investigación y Estudios Avanzados del Instituto Politécnico Nacional, México City, México; Baylor College of Medicine, United States of America

## Abstract

The effect of preventive human papillomavirus (HPV) vaccination on the reduction of the cervical cancer (CC) burden will not be known for 30 years. Therefore, it’s still necessary to improve the procedures for CC screening and treatment. The objective of this study was to identify and characterize cellular targets that could be considered potential markers for screening or therapeutic targets. A pyramidal strategy was used. Initially the expression of 8,638 genes was compared between 43 HPV16-positive CCs and 12 healthy cervical epitheliums using microarrays. A total of 997 genes were deregulated, and 21 genes that showed the greatest deregulation were validated using qRT-PCR. The 6 most upregulated genes (*CCNB2, CDC20, PRC1, SYCP2, NUSAP1*, *CDKN3*) belong to the mitosis pathway. They were further explored in 29 low-grade cervical intraepithelial neoplasias (CIN1) and 21 high-grade CIN (CIN2/3) to investigate whether they could differentiate CC and CIN2/3 (CIN2+) from CIN1 and controls. *CCNB2*, *PRC1*, and *SYCP2* were mostly associated with CC and *CDC20*, *NUSAP1*, and *CDKN3* were also associated with CIN2/3. The sensitivity and specificity of *CDKN3* and *NUSAP1* to detect CIN2+ was approximately 90%. The proteins encoded by all 6 genes were shown upregulated in CC by immunohistochemistry. The association of these markers with survival was investigated in 42 CC patients followed up for at least 42 months. Only *CDKN3* was associated with poor survival and it was independent from clinical stage (HR = 5.9, 95%CI = 1.4–23.8, p = 0.01). *CDKN3* and *NUSAP1* may be potential targets for the development of screening methods. Nevertheless, further studies with larger samples are needed to define the optimal sensitivity and specificity. Inhibition of mitosis is a well-known strategy to combat cancers. Therefore, *CDKN3* may be not only a screening and survival marker but a potential therapeutic target in CC. However, whether it’s indispensable for tumor growth remains to be demonstrated.

## Introduction

The human papilloma virus (HPV) is the main causal factor for the development of invasive cervical cancer (CC), and HPV is found in nearly 100% of these tumors [1], [2]. CC results from the progression of preinvasive cervical intraepithelial neoplasia (CIN), which is histologically graded into mild (CIN 1), moderate (CIN 2), or severe (CIN 3) dysplasia. CC occurs mainly from CIN3 and CIN2, but rarely from CIN1; the estimated progression rates of these lesions to CC are 12%, 5% and 1%, respectively [3]. Currently, there are vaccines on the market that prevent infection by oncogenic HPV types 16 and 18, which are associated with 65–70% of CCs worldwide [4]. These vaccines have very high efficiency for the prevention of infection and the development of high-grade cervical intraepithelial neoplasias (CIN2/CIN3) [5], [6]. However, vaccinated women must still attend programs for early detection of CC since these vaccines only protect against certain virus types, and it is not yet known how long the immune protection against the target virus remains [7], [8]. In many countries preventive vaccines for HPV 16 and 18 have been incorporated into the national vaccination program, for girls from 9 to 12 years of age [9], [10]. However, because the peak incidence of CC occurs in women 45–50 years old, the effect of these preventive vaccination programs on reducing the prevalence of CC will not be known for 30 years. Therefore, it is necessary to improve the procedures for CC screening and treatment. Because each year 530 000 new cases of CC and 275 000 CC deaths are reported worldwide, the incidence to mortality ratio is approximately 50% [11], [12].

For many years, the Papanicolaou (Pap) test has been the most important screening procedure for early detection of CC, and its massive application in developed countries has decreased the incidence of CC by more than 50% in the last 40 years [13]. Women with abnormal Paps are referred for colposcopy to confirm, discard, or clarify the diagnosis with a histopathological study. However, the average sensitivity of cytology for detection of CIN lesions is 50–60%; although the specificity is very high, approximately 90% [14]. Since HPV is indispensable for the development of CC, several procedures to detect the HPV genome have been incorporated into CC screening. Compared with conventional cytology, HPV DNA testing has higher sensitivity but lower specificity for the detection of CIN2 lesions or higher (CIN2+). The high sensitivity and high negative predictive value (NPV) of HPV DNA tests for the detection of CIN2+ lesions suggest that it could be used to extend screening intervals. However, the low specificity of HPV DNA tests would increase the number of follow-up tests and colposcopy referrals, which would increase the cost of screening [15]. Therefore, the need to develop new methods for early detection of CC with high sensitivity and specificity is clear. Multiple tumor markers associated with CIN2+ have been identified, especially *CDKN2A*, *TOP2A*, and *MCM2*. However, these markers have been proposed not for screening, but for diagnosis, prognosis, or clinical management [11], [16].

Invasive cervical cancer is currently treated with surgery, chemotherapy, radiotherapy, or a combination of these therapies, depending on the clinical stage of the disease. The success of these conventional therapies and patient survival diminishes as the disease progresses to more advanced stages [17]. In fact, the percentage of women who survive 5 years decreases from 93% for stage IA to 15% for stage IVB (www.cancer.org). In contrast to other types of cancer, for which several specific molecular drugs have been developed [18], there are no specific molecular-targeted therapies for CC. The majority of drugs against specific targets in cancer are directed toward mutated proteins, especially protein kinases [19]; however, some drugs target normal proteins that are overexpressed, such as HER2/neu in breast cancer [20], [21]. The first step in developing a specific molecular drug is identifying universal molecular targets that are present in patients with CC and absent in healthy women.

The objective of this study was to identify and characterize cellular targets present in most CCs and absent from normal cervical tissue that differ enough between the 2 groups to be considered either as potential markers for screening, with a sensitivity and specificity close to 100%, or as potential therapeutic targets.

## Methods

### Ethics Statement

The study protocol was approved by the Scientific and Ethics Committees of the Hospital General de Mexico (approval number DIC/03/311/04/051) and was performed in accordance with the ethical principles described in the 1964 Declaration of Helsinki. Informed written consent was obtained from all participants prior to their inclusion in the study.

### Subjects, Samples, and Experimental Design

The study subjects included 69 patients with invasive cervical cancer (CC) diagnosed in the Department of Oncology, 29 patients with low-grade CIN (CIN1), 21 patients with high-grade CIN (CIN2 and CIN3), and 25 women with normal cervical epithelium evaluated in the Department of Obstetrics and Gynecology at the Hospital General de México in Mexico City. The CC samples were a subset selected from a total of 462 patients with CC who were recruited sequentially from November 2003 through July 2007, which represented approximately 80% of the patients newly diagnosed with CC during this period due to the restrictive inclusion criteria (no previous treatment, incident case, born in Mexico with Mexican ancestry for 2 generations). The selection criteria for the CC subset were based on the availability of a fresh tumor biopsy for RNA extraction with more than 70% tumor cells in the morphological analysis (see below), mostly FIGO stages I/II, and viral type. This subset included 47 samples positive for HPV16 and 22 samples positive for other virus types, including HPV18, 31, 33, 45, 51, 58, and 59. Among them, 54 samples were of squamous cell carcinomas, 14 samples were of adenocarcinomas, and 1 sample was of an adenosquamous carcinoma. The average age of patients with cancer was 48 years (range, 23–78 years; Table S1). All patients received complete clinical evaluations. The tumors of CC patients were staged according to the last international revised protocol for gynecologic cancer [22]. One or two biopsies, conducted under colposcopy examination, were taken from tumors. One biopsy was divided in 2 equal parts, 1 part was fixed in buffered formol for morphological analysis and the other part, together with the second biopsy, was snap-frozen on dry ice and stored at −80°C until analysis. All CC patients were referred for surgery, radiation, chemotherapy, or a combination of these treatments according to the guidelines of the American Cancer Society (see below). Control cervical specimens were obtained from patients undergoing hysterectomy due to myomatosis at the Gynecology Service of the Hospital General de Mexico. They were previously diagnosed with a normal cervix by cytology and colposcopy. Immediately after receiving a cervix fragment from the operating room, the exocervical and endocervical epitheliums were dissected under a stereoscopic microscope to avoid the stromal cells. The tissues were then snap frozen in liquid nitrogen and stored at −80°C until use. For HPV detection and typing, a scrape from the endocervix and ectocervix was collected with a cytobrush from the patients and controls, the cells were suspended in a vial with extraction buffer, and then stored at −20°C until analysis. Analysis of global gene expression (8,638 genes) was performed in RNAs extracted from 43 fresh tumor biopsies positive for HPV16 and from 12 samples of normal cervical epithelium using the HG-Focus microarray. Global gene expression was validated in 24 samples, including 19 CCs and 5 cervical epithelium controls, by a second high throughput microarray (HG-ST1.0). The 23 genes that showed the greatest deregulation were validated by real time PCR (qRT-PCR) in 44 HPV16-positive CC and 25 control samples. The 6 most differentially expressed genes (*CCNB2*, *CDC20*, *PRC1*, *SYCP2*, *NUSAP1*, and *CDKN3*) were further explored in 29 low-grade cervical intraepithelial neoplasias (CIN1) and 21 high-grade CIN (CIN2/3) to investigate whether they could differentiate CC and CIN2/3 (CIN2+) from CIN1 and controls. Immunohistochemistry (IH) was performed for 10 selected proteins in 26 CC samples and 10 control samples. The association of 9 markers with survival was investigated by survival analysis of 42 patients with HPV16-positive CC who were followed up for at least 42 months.

### DNA and RNA Isolation

DNA was purified from cervical scrapes and some biopsy specimens using the PureLink Genomic DNA Kit (Invitrogen, Grand Island NY) and maintained at −20°C until analysis. Total RNA was isolated from one half of the divided biopsy using TRIzol reagent (Invitrogen), according to the manufacturer’s protocol. The quality of the RNA was confirmed by agarose gel electrophoresis, as demonstrated by the presence of intact ribosomal RNA, with the 28s band twice as intense as the 18s band.

### Detection and HPV Typing

HPV detection was performed by PCR using universal primers located in the HPV *L1* gene *MY09*/*MY11*, *GP5*+/*6+*, and *L1C1* as described previously [23]–[25]. The *HBB* gene was used as an internal control to assess the quality of DNA. The HPV types were identified by sequencing the amplified bands in positive samples using a fluorescent cycle-sequencing method (BigDye Terminator Ready Reaction Kit, Applied Biosystems, Foster city, CA). Sequence analysis was performed using an ABI PRISM 3130xl genetic analyzer (Applied Biosystems). Each sequence from the HPV positive samples was analyzed with the FASTA sequence similarity tool [26]. The average percentage identity of these sequences to HPV types was 98.7% (range, 91–100%).

### Gene Expression Profiling and Data Analysis

The gene expression profile of 43 CCs positive for HPV16 and 12 healthy control cervical epitheliums was examined using the Human Gene Focus (HG Focus) oligonucleotide Microarray (MA) (Affymetrix, Santa Clara, CA). This array contains 8,794 probe sets corresponding to 8,638 characterized human genes in the Gene Reference database. Total RNA preparation (10 µg), labeled cRNA synthesis, hybridization, scanning, and image analysis were performed according to the manufacturer’s protocols (Affymetrix GeneChip Expression Assay manual). To assess the quality of the experiments, the following parameters were used: increased expression of exogenous poly-A controls (Lys<Phe<Thr<Dap), the presence of oligo B2 used to make grid alignments, background with an acceptable range of 20 to 100, equal noise across all samples, percentage of present calls greater than 50%, a 3′/5′ ratio of a constitutive gene (GAPDH or β-actin) of less than 3, and increased expression of the hybridization controls (BioB<BioC<BioD<cre). Only those MAs with optimal quality controls were analyzed. Furthermore, some samples were performed in duplicate to evaluate the reproducibility of the experiment, which was higher than 99%.

MA intensity values were normalized using the Robust Multichip Average (RMA) algorithm using FlexArray software [27]. The normalized intensity values were referred to as units of intensity (UI). Genes expressed differently between the tumors and controls were identified using the algorithm Significance Analysis of Microarrays (SAM version 3.0, http://www.stat.stanford.edu/~tibs/SAM) using the cut-off values of a fold change (FC) of ≥1.5, a general false discovery rate (FDR) of 1%, and a local FDR of <10% [28]. Unsupervised hierarchical clustering and principal component analysis (PCA) were performed using dChip software (version 1.6, www.dCHIP.org) and R language in Java’s platform, respectively.

### Validation of Global Gene Expression by a Second High throughput Microarray (HG-ST1.0)

The gene expression profile of 24 samples explored with the HG-Focus microarray, including 19 CCs and 5 cervical epithelium controls, was also examined using the Human Gene 1.0 ST oligonucleotide microarray (Affymetrix, Santa Clara, CA). This array contains 33,297 probe sets that correspond to approximately 20,741 genes of the human gene reference database according to the UCSC Genome Browser Assembly Mar. 2006 NCBI 36/hg18, available at http://genome.ucsc.edu/. Total RNA preparation (300 ng), labeled DNA synthesis, hybridization, scanning, and image analysis were performed according to the manufacturer’s protocols (Affymetrix GeneChip Expression Assay manual). To assess the quality of the experiments, the following parameters were used: expression of the exogenous poly-A controls, the presence of oligo B2 used to make grid alignments, and area under the curve (AUC) values above 0.8. Only those microarrays with optimal quality controls were analyzed. Microarrays were normalized using the RMA algorithm in the Affymetrix expression console. The normalized intensity values were referred to as units of intensity (UI). The normalized intensities (log_2_ values) of the 8,370 genes that were examined on both microarrays (HG ST1 and HG Focus) were compared, and the level of correlation was assessed with Pearson’s correlation coefficient.

### Validation of Global Gene Expression by Real-time Quantitative Retrotranscription PCR (qRT-PCR)

Reverse transcription of total RNA was performed using the High-Capacity cDNA Archive kit (Applied Biosystems) in a total volume of 20 µL. The mix included 2 µg of RNA, 2 µL of 10× RT buffer, 0.8 µL of 100 mM dNTPs, 2 µL of 10× RT Random Primers, 1 µL of MultiScribe™ reverse transcriptase (5 U/µL), and 1 µL of RNase inhibitor (2 U/µL). Reactions were incubated at 37°C for 120 min, and then stored at −20°C. A set of 23 genes was used to validate gene expression in 44 HPV16-positive CC and 25 healthy cervical epithelium control samples with qRT-PCRs using TaqMan probes. The genes included are *CCNB2, CDC2, CDC20, CDKN2A, CDKN3, CKS2, MCM2, MKI67, NUSAP1, PCNA, PRC1, RFC4, RRM2, SMC4, SYCP2, TOP2A, TYMS, ZWINT, CFD, EDN3, NDN, SLC18A2,* and *WISP2*. *GAPDH* was used as internal control. TaqMan gene expression assays were used (Table S2; Applied Biosystems). Seven genes were also explored in 22 CC positive for other HPVs (*CCNB2*, *CDC20*, *CDKN3*, *PRC1*, *SYCP2*, *NUSAP1*, *TYMS*), and the first 6 of them, along with *CDKN2A*, *PCNA*, *MKI67* genes, were further explored in 29 low-grade CINs and 21 high-grade CINs. The experiments were run in duplicate in a final volume of 20 µL, including 200 ng of cDNA template, 10 µL of 2× TaqMan Universal PCR Master Mix (Applied Biosystems), 1 µL of 20× TaqMan Gene Expression Assay, and 7 µL of RNase-free water. The cycling program was run in a Rotor-Gene (Corbett Research, Sydney, Australia), which was set as follows: an initial PCR activation step at 50°C for 2 min followed by 95°C for 10 min, then 40 cycles of melting at 95°C for 15 s and annealing/extension at 60°C for 1 min. The median of the Ct standard deviations in duplicates ranged from 0.09 to 0.24 (mean = 0.16) among the 23 genes, suggesting that the variations between the duplicates were very small [29]. Measurement of gene expression was based on relative standard curves constructed from a 10-fold serially diluted pool of CC or normal cervical epitelium cDNAs ranging from 500 to 0.05 ng. The first curve was used to calculate the values of upregulated genes and the second curve the values of downregulated genes. Curves for each gene were tested in three different experiments ran in duplicate and the averages of the correlation coeficients (r) were higher than 0.98. The expression of target genes was normalized in each tumor and control sample to the intensity of the internal reference (*GADPH*) using a previously described method [30]. The normalized intensity values were measured in ng/µL. A normality test (Shapiro-Wilk) was carried out to test for a normal distribution of gene expression data. The fold-change expression was calculated by dividing the median normalized intensity of tumor samples by the median normalized intensity of the control samples. The statistical significance between the medians of tumors and controls was calculated with the Mann–Whitney (MW) non-parametric test. The correlations between the MA results and the qRT-PCR data were performed using log_2_ values and measured using Pearson’s correlation coefficient.

### Immunohistochemistry

The protein expression of 10 genes was determined in 26 CC and 10 control samples with IH. Two homemade tissue microarrays (TMA) were built, one containing 14 HPV16-positive CCs and 5 controls and the other 12 CC positive for other HPVs and 5 controls. NUSAP1 was explored only in samples of the first TMA. Cylindrical samples from representative regions of the paraffin embedded tissue blocks, previously selected by H&E stained slides, were taken with a punch-biopsy needle (2 mm diameter), transferred to recipient paraffin blocks in defined array positions and newly embedded in paraffin. All the tissues blocks of matched patients were obtained from the Pathology Department of the hospital. Serial sections (4 µm thick) of the TMA were cut and the 10th slide was stained with H&E to confirm the histopathological diagnosis. Sections were immersed in xylene to remove paraffin and then rehydrated with graded alcohol (100%, 95%, 90%, 80%, and 70% v/v in water). Epitope retrieval was performed by heating the slides, and introducing them into Target Retrieval Solution, pH 6.0 (Dako, Carpinteria, CA) at 121°C for 5 min in a pressure cooker. Endogenous peroxidase activity was blocked by incubating the slides with 1% hydrogen peroxide in PBS for 10 min. Then, a non-specific background blocker was added and incubated for 10 min. Primary antibodies against PCNA (sc-53407); p16 for CDKN2A (sc-71804); SCP-2 for SYCP2 (sc-20048), PRC1 (sc-56345); cyclin B2 for CCNB2 (sc-81241); CDKN3 (sc-475); and CDC2 for p34 (sc-70822), were obtained from Santa Cruz Biotechnology (Santa Cruz, CA). The antibodies against CDC20 (cat. 34–1900), Ki-67 for MKI67 (cat. M7187) and NUSAP1 (cat. H00051203-B01) were obtained from Invitrogen, Dako (Glostrup, Denmark), and Nova Biological (Littleton, CO), respectively. The dilution used for all antibodies was 1∶100, except for CDC2, (1∶50) and NUSAP1 (1∶250), and the antibody diluent used was from Dako. A total volume of 300 µL was added to each section, and the slides were incubated overnight at 4°C in a moist chamber. Antigen-antibody complexes were detected by the avidin-biotin peroxidase method, using 3,3′-diaminobenzidine-tetrahydrocloride as a chromogenic substrate (Cat. KO679 LSAB+Sys/HRP; Dako-Cytomation Carpinteria, CA), and the sections were counterstained with hematoxylin. Assays were performed in triplicate. The antibodies for SYCP2, PRC1, CCNB2, CDKN3, CDC2, and CDC20 were tested in tissues known to express those antigens. SYCP2 was tested in neonate testis; PRC1, CDC2, and CCNB2 were tested in colon cancer; and CDKN3 was tested in lung cancer biopsies. All tissues were obtained from the archives of the Pathology Department. The percentage of stained cells was calculated from an analysis of 10 successive high-power fields of neoplastic cells. The cellular localization of the immunoreaction was identified, and the intensity of the immunoreaction was scored from 0 to 4, where 0 indicated no staining. Immune reaction signals were found rarely in the stroma with all antibodies and were not scored for the analysis. Immunostained slides were analyzed and scored by 2 pathologists, who were blinded to the outcomes. Rare cases with discordant scores were reevaluated and scored based on consensus opinion.

### Survival Analysis of Cancer Patients

According to FIGO staging patients with cervical cancer received individualized treatment based on the treatment guidelines for cervical cancer of the American Cancer Society (See Table 1). After the treatment was completed, each patient was clinically evaluated every 3 or 6 months by an experienced oncologist. Clinical data of the follow-up study was obtained from the patients medical record. Also, a social worker performed phone calls and home visits to the patients every 6 months during the study. Patients recorded as alive in the study were successfully followed up for at least 42 months after treatment. Censored and deceased patients were followed up for the number of months indicated in Table 1. The cases designated as censored referred to those patients who were lost to the study in the follow-up period or deceased from causes other than cervical cancer. Patients were considered lost when did not attend to medical appointments for disease control, were not found at home visits or did not answer phone calls. In this cohort, patients recorded as deceased were only those women who died by cervical cancer primary tumor as a main cause. The cause of death of all but one patient who died during the follow up was confirmed by the medical record and the death certificate. Only 42 of 44 patients with HPV16-positive CC explored with qRT-PCR were included in the followed up study. Four cases were considered right censored and eight deaths were registered. The mean following time of the 42 patients was 50.5 months. The association of FIGO and gene expression (*PRC1, CCNB2, CDC20, CDKN3, NUSAP1, SYCP-2, CDKN2A, PCNA, MKI67*) with survival was investigated by survival analysis. With the whole sample set, 500 training sets of 21 samples were randomly created for each gene explored. To categorize the gene expression data quantified by qRT-PCR, ROC analysis was performed in each training set. This analysis was done to set a cut-off for gene expression that represented those values with the highest sensitivity and specificity to differentiate between dead and surviving patients. The whole sample set was then analyzed with the average cut-off, calculated from the values of the 500 training sets. Samples with gene expression values above the cut-off were set to 1 and those with values below the cut-off were set to 0. The cumulative overall survival time was calculated by the Kaplan-Meier method and analyzed by the log-rank test. FIGO staging and the gene expression were included as covariates in a Cox proportional hazard model.

**Table 1:** Patients followed up for at least 42 months for survival evaluation.

Sample	Histology^a^	Tumor Stage	Age (years)	Treatment^b^	Follow up (months)	Status^c^
R093	ACC	IB1	57	HT	53	Alive
R446	ACC	IB1	43	HT	59	Alive
R081	ACC	IB1	41	HT	62	Alive
R094	SCC	IB1	45	HT	62	Alive
R369	SCC	IB1	50	HT	65	Alive
R057	ACC	IB1	32	HT	93	Alive
R072	SCC	IB1	61	HT+TELE+BRACHY	86	Alive
R411	SCC	IB1	34	HT+TELE+BRACHY	60	Alive
R434	ACC	IB1	34	HT+TELE+BRACHY	61	Alive
R443	SCC	IB1	34	HT+TELE+BRACHY	61	Alive
R258	SCC	IB1	36	HT+TELE+BRACHY	68	Alive
R335	ACC	IB1	37	HT+TELE+BRACHY+CHEMO	65	Alive
R183	SCC	IB1	64	TELE+BRACHY	61	Alive
R308	ACC	IB1	45	TELE+BRACHY	61	Alive
R265	SCC	IB1	46	TELE+BRACHY	67	Alive
R330	SCC	IB1	72	TELE+BRACHY	54	Alive
R035	SCC	IB2	48	TELE+CHEMO+HT	73	Alive
R221	SCC	IB2	41	TELE+BRACHY+CHEMO	33	Death*
R232	SCC	IB2	45	TELE+BRACHY+CHEMO	33	Death
R409	SCC	IB2	68	TELE+BRACHY+CHEMO	42	Alive
R339	SCC	IB2	31	TELE+BRACHY+CHEMO	13	Death
R324	SCC	IB2	28	TELE+BRACHY+CHEMO	14	Death
R359	ACC	IB2	34	TELE+BRACHY+CHEMO	17	Death
R378	SCC	IB2	42	TELE+BRACHY+CHEMO	56	Alive
R396	ACC	IB2	53	TELE+BRACHY+CHEMO	7	Unknown
R312	ACC	IB2	34	TELE+BRACHY+CHEMO	58	Alive
R482	SCC	IB2	61	TELE+BRACHY+CHEMO	60	Alive
R284	ACC	IB2	33	TELE+BRACHY+CHEMO	63	Alive
R412	SCC	IB2	33	TELE+BRACHY+CHEMO	63	Alive
R336	SCC	IB2	36	TELE+BRACHY+CHEMO	64	Alive
R255	SCC	IIA	45	TELE+BRACHY+CHEMO	42	Death
R052	ACC	IIB	54	TELE+BRACHY+HT	19	Death
R070	SCC	IIB	74	TELE+BRACHY+CHEMO	4	Unknown
R170	SCC	IIB	67	TELE+CHEMO+HT	82	Alive
R403	SCC	IIB	34	TELE+BRACHY+CHEMO	64	Alive
R015	SCC	IIB	42	TELE+HT	66	Alive
R268	SCC	IIB	34	TELE+BRACHY+CHEMO	58	Alive
R415	ASCC	IIB	55	TELE+BRACHY+CHEMO	59	Alive
R441	ACC	IIB	24	TELE+BRACHY+CHEMO	10	Unknown
R333	SCC	IIB	56	TELE+BRACHY+CHEMO	66	Alive
R315	SCC	IIIB	41	TELE+BRACHY+CHEMO	8	Death
R240	SCC	IIIB	31	TELE+BRACHY+CHEMO	11	Death

a ACC, Adenocarcinoma. SCC, Squamous Cell Carcinoma. ASCC, Adenosquamous Cell Carcinoma.

b HT, Radical Hysterectomy. Tele, teletherapy. Brachy, brachytherapy. Chemo, chemotherapy with Cisplatin.

c Status alive was registered at the last follow up, death was caused by primary tumor of cervical cancer, except the case labeled with an asterisk, and unknown cases were lost during the follow up study. The cause of death of case labeled with an asterisk was unknown.

### Gene Ontology Classification Analysis

The Database for Annotation, Visualization, and Integrated Discovery (DAVID) functional annotation tool (http://david.abcc.ncifcrf.gov) [31], [32] and the Ingenuity Pathway Analysis (IPA; Ingenuity® Systems, www.ingenuity.com) were used to classify the deregulated genes. Genes were classified using functional annotation clustering considering the gene ontology biological processes. Classification stringency was set at medium and maximum level.

### Gene Annotation and Data Analysis

The physical position of genes was mapped according to the UCSC Genome Browser Assembly Mar. 2006 NCBI 36/hg18, available at http://genome.ucsc.edu/. Data analysis was performed using Access 2010 (Microsoft Inc.). The raw MA data is MIAME compliant and has been deposited in a MIAME compliant database (GEO, http://www.ncbi.nih.gov/geo/) under the accession number GSE39001. Receiver operator characteristic (ROC) curve analysis was performed and Youden index was used [33] to select the best cut-off points to distinguish tumors from controls and CIN2+ from CIN1− using the expression values of selected genes obtained by qRT-PCR. For each marker, the sensitivity, specificity, positive predictive value (PPV), and negative predictive value (NPV) were calculated according to previously described formulas [34]. All tests were 2 sided, and p-values less than 0.05 were considered statistically significant. Data analysis was performed using Sigma Stat and SPSS ver. 17 software.

## Results

### Expression Analysis of 8,638 Genes in Cervical Cancer

**Figure 1:**
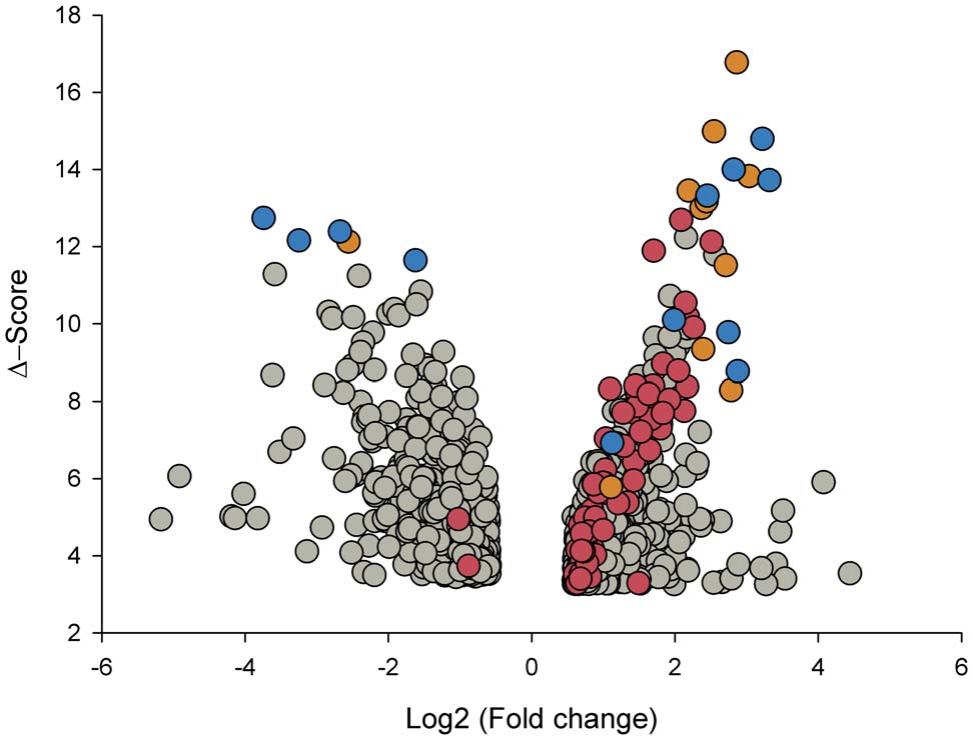
Distribution of deregulated genes according to the fold change (FC) and Δ-score values. All 997 genes (circles) that were deregulated in cervical cancer (CC) tumors compared to the control samples by the SAM method are graphed in a Volcano plot. The x-axis represents the FC in gene expression (cancer sample/control sample) expressed in Log2 and the y-axis display the absolute Δ-score, a modified t-test calculated with the SAM method, the higher the Δ-score values, the higher the statistical significance. The Log2 (FC) values are positive for upregulated genes and negative for downregulated genes. Circles colored in red and orange represent the genes involved in M-phase of the cell cycle and those colored in blue and orange are the genes that were validated by qRT-PCR.

**Figure 2:**
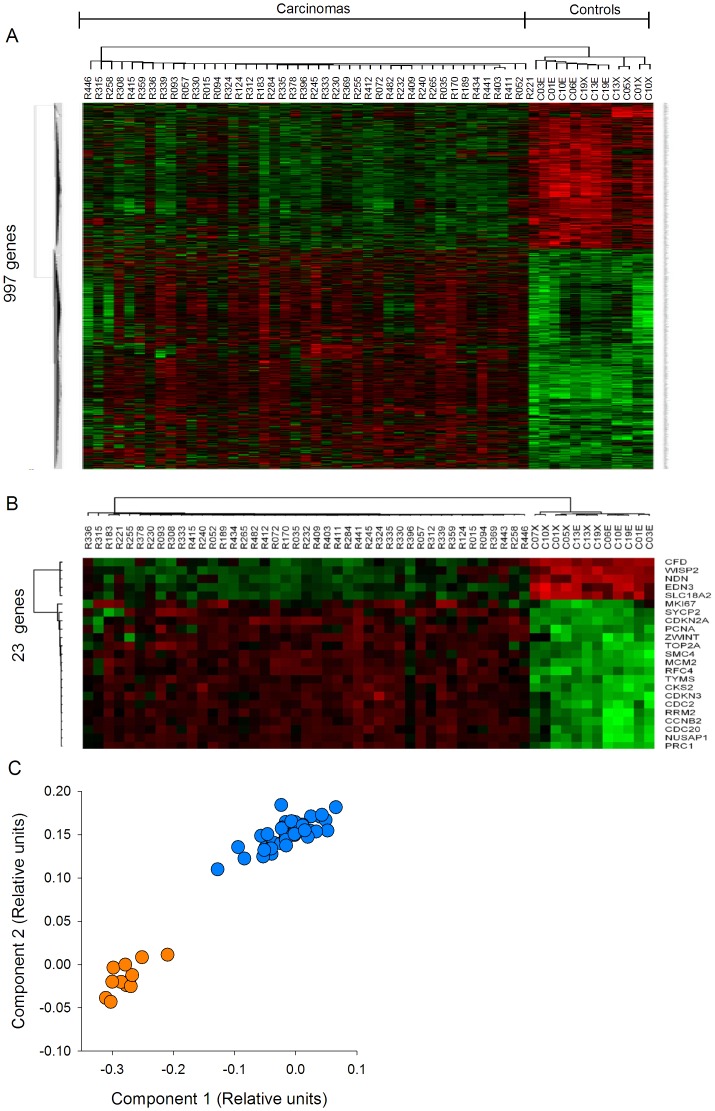
Segregation of tumor and control samples according to the expression of deregulated genes. Unsupervised hierarchical cluster analysis of 43 CCs and 12 healthy cervical epitheliums using the expression values obtained with the HG-Focus microarray of all 997 deregulated genes (panel A) or the 23 top ranked genes selected for validation (panel B). Each row represents a gene and each column represents a sample. The length and the subdivision of the branches represent the relationships among the samples based on the intensity of gene expression. The cluster is color-coded using red for upregulation, green for downregulation, and black for unchanged expression. Panel C shows the principal components analysis (PCA) using the values in panel B; blue circles represent the CCs (n = 43) and yellow circles represent the controls (n = 12). Both sets of genes clearly separated the samples into the 2 main groups using both types of analysis.

The amount of mRNA transcribed from 8,638 genes was compared between 43 CC samples positive for HPV16 and 12 normal cervical epithelial samples using the HG-Focus microarray. A total of 997 genes were differentially expressed between the cancer and control groups; 600 were upregulated and 397 were downregulated (Table S3). Almost one-half of the upregulated and downregulated genes had FCs in the range of 1.5–2.0, and the number of genes in both groups decreased linearly (r = −0.8, p = 0.002) as the FC value increased (Figure 1). The principal component analysis (PCA; data not shown) and the non-supervised hierarchical clustering (panel A in Figure 2) performed with all 997 gene expression values clearly separated the cancer samples from the control group. However, the expression of many genes was not completely uniform among the cancer samples, especially in the group of upregulated genes (signals shown in red in Figure 2A). Many of those genes were upregulated in some tumors and downregulated in other tumors. This was in contrast to the uniformity of the expression signals in the control group samples. Genes to be tested as markers for screening or as potential therapeutic targets were selected according to Δ-score rank (a modified t-test, used in SAM), FC or whether they were previously used as markers for cervical cancer. From the 997 genes associated with the cancer samples, 163 have been previously reported as markers for different types of cancer (IPA, Ingenuity Systems), including *MCM2*, *TOP2A*, and *CDKN2A*, which have been used as markers for diagnosis in cervical cancer [35]. The 997 genes were listed in decreasing ordered by Δ-score (Table S3). A total of 23 genes (18 upregulated and 5 downregulated) were selected for validation by qRT-PCR (marked in bold in Table S3 and Table 2; circles colored in blue and orange in Figure 1). All downregulated genes (*CFD*, *NDN*, *WISP2*, *END3*, and *SLC18A2*) and 10 of the 18 upregulated genes (*PRC1*, *CKS2*, *TYMS*, *RFC4*, *RRM2*, *NUSAP1*, *MCM2*, *CCNB2*, *SMC4*, and *CDC2*) were selected according to Δ-score rank. Seven of the remaining upregulated genes are on the list of the 50 best ranked genes, 2 of them are genes that have been previously proposed as markers in CC (*CDKN2A* and *TOP2A*), 4 (*CDC20*, *CDKN3*, *ZWINT*, and *SYCP2*) were selected based on the FC value, and *PCNA* (Table S3), together with *MKI67*, which ranked in 139th place, were included because these markers are commonly used to measure cell proliferation. The PCA analysis and hierarchical clustering showed that the 23 selected genes also allowed for segregation of the samples into the 2 different groups. For both the upregulated and downregulated genes, the difference in signal intensities was quite uniform among the samples from the 2 groups (Figure 2, panels B and C).

**Table 2:** Genes explored by qRT-PCR.

	Fold change^b^	
Gene^a^	HPV16 positive	Other HPVs^c^
Upregulated		
***MKI67***	1651	–
***CDKN2A***	387	–
***SYCP2***	74	14
***PCNA***	65	–
***NUSAP1***	26	15
*CDC2*	23	–
***CDC20***	17	13
***CCNB2***	14	6
*TYMS*	12	2
***PRC1***	9	4
*SMC4*	8	–
***CDKN3***	7	5
*RRM2*	6	–
*CKS2*	5	–
*MCM2*	4	–
*ZWINT*	4	–
*RFC4*	4	–
*TOP2A*	3	–
Downregulated		
*EDN3*	1426	–
*WISP2*	168	–
*CFD*	25	–
*NDN*	1	–
*SLC18A2*	0.3	–

a Genes in bold were selected to be explored in pre-invasive samples.

b The analysis was performed with 44 HPV16-positive CC, 22 CC positive for other HPVs and 25 cervical controls. Fold change (FC) was calculated with the median values as follows: tumor/control for upregulated genes and control/tumor for downregulated genes (see Materials and Methods). The difference between the groups was statistically significant (p<1×10^−15^; Mann-Whitney Rank Sum Test) for all but 2 genes (NDN, SLC18A2). NDN and SLC18A2 had a p>0.05.

c Included carcinomas positives for HPV-18 (5), -31 (5), -33 (2), -45 (5), -51 (2), -58 (2) and -59 (1).

### Validation of Genes with HG-ST1.0 Microarray and Quantitative Real-time PCR (qRT-PCR)

**Figure 3:**
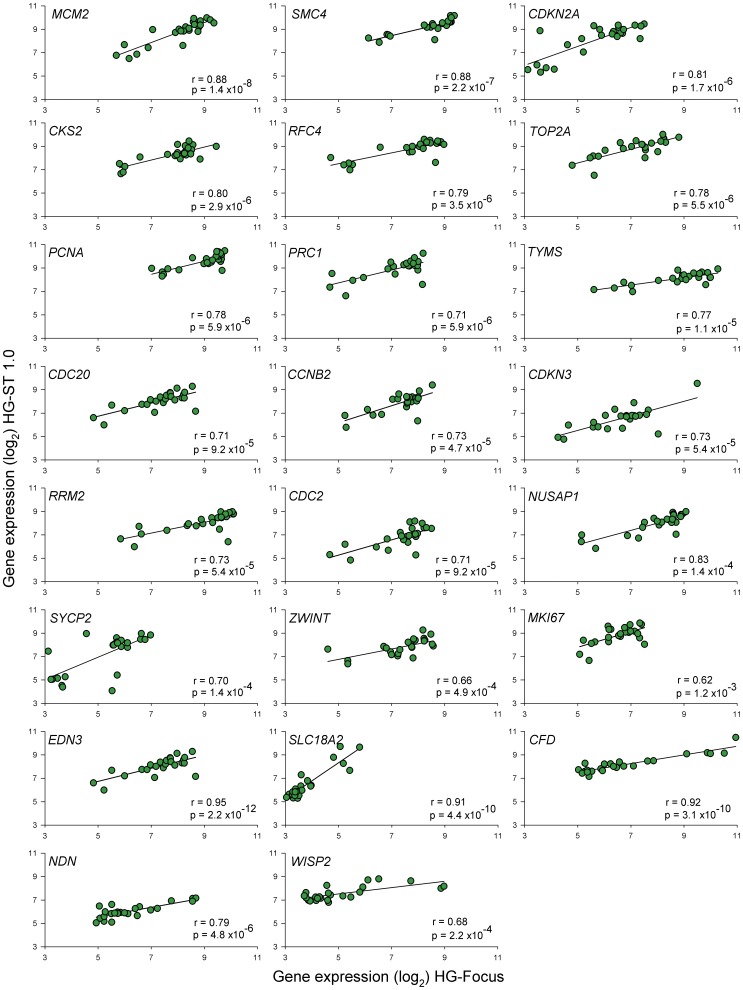
Correlation of expression intensity of 23 genes examined by HG-Focus and HG-ST1.0 microarrays. The Log_2_ values of the standardized intensity signals (RMA values) of 23 genes examined by the 2 microarrays in 19 CC and 5 normal cervical epithelium were plotted. The linear trend (black line) is included, which was calculated with Person’s correlation test. r =  correlation coefficient, p =  p-value.

A total of 8,370 genes were validated with microarray HG-ST1.0 in 24 samples explored with the HG-Focus microarray, including 19 CC samples and 5 healthy cervical epitheliums. Highly significant positive correlations (p<1×10^−15^, Pearson’s correlation) were found between the HG-ST1.0 and HG-Focus microarray values. The global correlation between the 2 arrays was 0.68 and the correlation coefficients between the individual tumors ranged from 0.57 to 0.72 (average, 0.68). Gene expression values of 826 out of 997 (82.8%) genes expressed differently between the cancer and control samples, including the 23 genes selected for validation (Figure 3), showed significant positive correlations (p<0.05, Pearson’s correlation) between the 2 microarrays. The correlation coefficients between the individual genes ranged from 0.34 to 0.95 and the average was 0.63 (Figure S1).

**Figure 4:**
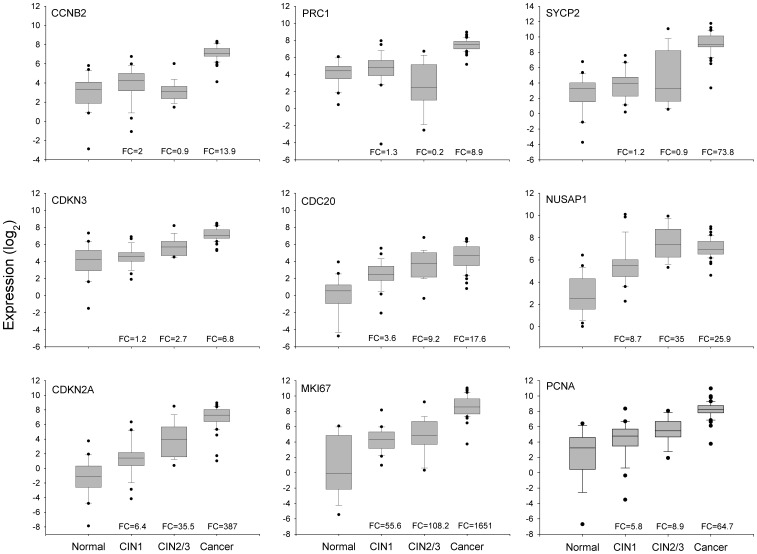
Validation of gene expression of 9 genetic markers by qRT-PCR. The intensity of gene expression, expressed in Log2 values, is shown in box plots. Expression of the 6 genes validated in this study (*CCNB2*, *PRC1*, *SYCP2*, *CDKN3*, *CDC20*, and *NUSAP1*) and the 3 well-known genes (*CDKN2A*, *MKI67*, and *PCNA*) associated with CC are compared among the 4 groups, including healthy cervical epitheliums (Normal, n = 25), low-grade CIN (CIN1, n = 29), high-grade CIN (CIN2/3, n = 21), and invasive CC (cancer, n = 44). The upper and lower boundaries of the boxes represent the 75^th^ and 25^th^ percentiles, respectively. The black line within the box represents the median value, and the whiskers represent the minimum and maximum values that lie within 1.5× the interquartile range from the end of box. Values outside this range are represented by black circles. The fold change (FC) was calculated by dividing the median of each pathological group by the median of the control group.

On the other hand, the expression of the 23 genes selected for validation was measured with qRT-PCR in a total of 44 HPV16-positive cancer samples and 25 healthy cervical epitheliums, including almost all samples previously determined with MA (Table S1). A highly significant positive correlation (p<0.0001, Pearson’s correlation) was found between the qRT-PCR and MA log_2_ values in 21 of the 23 measured genes. The correlation coefficients ranged from 0.31 to 0.85 and the median was 0.73. The 2 genes that had non-significant correlations (*NDN* and *SLC18A2*) were excluded from the rest of the analysis. These data indicated that the expression values calculated from the microarrays were fairly reliable because 91% of validated genes had a significant correlation. Since the qRT-PCR expression values of 87% of the measured genes did not follow a normal distribution, the median rather than the mean was used for the calculations of FCs. Genes were listed in decreasing order by the FC (Table 2) and at the top of this list is *MKI67*, which is followed in decreasing order by *CDKN2A*, *SYCP2, PCNA*, *NUSAP1*, and *CDC2*. It is worth noting that the FCs of *MKI67* (1,651), and *CDKN2A* (387) are at least 5 times higher than the FC of the gene that follows on the list (*SYCP2*; FC = 73.8). Of the top 10 ranked upregulated genes, 2 have not been previously reported as associated with cervical cancer (*NUSAP1*, and *CDKN3*), while the other 8 have been associated with cervical cancer either scantly (*SYCP2*, *PRC1*, *CCNB2* and *CDC20*) or widely (*MKI67*, *CDKN2A*, *CDC2*, and *PCNA*). *MCM2* and *TOP2A*, which have been widely reported as associated with cervical cancer, ranked 15th and 18th on the list, respectively. The 3 downregulated genes that had a significant Pearson’s correlation also had a high FC (controls vs. cancers), especially *END3* (FC = 1,425.7) and *WISP2* (FC = 167.7; Table 2). The box plots (Figure 4 and Figure S2) clearly show the difference in gene expression between the cancer and control groups (p<1×10^−15^ for all genes, Mann–Whitney *U* test).

To establish a separation line between the 2 groups and the potential value of these genes as markers of cervical cancer, cut-off values were established by analyzing ROC curves. In general, ROC curves with an area under the curve (AUC) ≤0.75 are not clinically useful, while an AUC of 0.97 has a very high clinical value [36]. The AUC of 11 upregulated genes (*CDKN2A*, *MKI67*, *PRC1*, *CDC2*, *CCNB2*, *SYCP2*, *PCNA*, *NUSAP1*, *TYMS*, *CDC20*, and *CDKN3*) and 1 downregulated gene (*CFD*) was ≥0.97 (Table 3). In fact, most of these genes had a sensitivity and specificity greater than 95%, suggesting that they could be good markers for screening between healthy samples and invasive cancers. Interestingly, this subset included 2 genes that were not reported to be associated with CC (*NUSAP1*, and *CDKN3*) and 5 genes that were scantly reported to be associated with CC (*PRC1*, *SYCP2*, *CCNB2*, *TYMS*, and *CDC20*). These 7 genes were examined with qRT-PCR in the 22 CC samples positive for other viral types, including HPV18, 31, 33, 45, 51, 58, and 59. All of them were upregulated in these 22 tumors; however, the FCs were lower than those obtained in the HPV16-positive tumors (Table 2). These data suggest that these 7 genes might be upregulated in all invasive CCs regardless of viral type; therefore, they could be considered potential markers for CC screening.

**Table 3:** ROC analysis and calculus of sensitivity, specificity and predictive values.

			Controls (n = 25)		Cervical Cancer (n = 44)							
Genes	AUC	Cut-off value^a^	FPF	TNF	TPF	FNF	*p*-value^b^	Sensitivity	Specificity	PPV	NPV	Youden index^c^
*CDKN2A*	0.996	18	0	25	42	2	<1×10^−10^	0.95	1	100	92.6	0.95
*CCNB2*	0.995	58	0	25	43	1	<1×10^−10^	0.98	1	100	96.2	0.98
*MKI67*	0.995	79	0	25	43	1	<1×10^−10^	0.98	1	100	96.2	0.98
*PRC1*	0.995	80	0	25	43	1	<1×10^−10^	0.98	1	100	96.2	0.98
*CDC2*	0.995	85	0	25	42	2	<1×10^−10^	0.95	1	100	92.6	0.95
*SYCP2*	0.992	115	0	25	42	2	<1×10^−10^	0.95	1	100	92.6	0.95
*NUSAP1*	0.990	48	1	24	43	1	<1×10^−10^	0.98	0.96	97.7	96.0	0.94
*PCNA*	0.990	100	0	25	42	2	<1×10^−10^	0.95	1	100	92.6	0.95
*TYMS*	0.985	46	0	25	41	3	<1×10^−10^	0.93	1	100	89.3	0.93
*CDC20*	0.971	3	3	22	42	2	<1×10^−10^	0.95	0.88	93.3	91.7	0.83
*CDKN3*	0.970	83	1	24	41	3	<1×10^−10^	0.93	0.96	97.6	88.9	0.89
*SMC4*	0.960	431	1	24	40	4	<1×10^−10^	0.91	0.96	97.6	85.7	0.87
*RFC4*	0.905	221	4	21	42	2	<1×10^−10^	0.95	0.84	91.3	91.3	0.79
*RRM2*	0.905	103	5	20	41	3	3×10^−9^	0.93	0.8	89.1	87.0	0.73
*TOP2A*	0.866	128	5	20	43	1	<1×10^−10^	0.98	0.8	89.6	95.2	0.78
*MCM2*	0.846	121	4	21	40	4	2.5×10^−9^	0.91	0.84	90.9	84.0	0.75
*ZWINT*	0.827	59	7	18	39	5	1.1×10^−6^	0.89	0.72	84.8	78.3	0.61
*CKS2*	0.815	239	5	20	35	9	5×10^−6^	0.80	0.8	87.5	69.0	0.60
			TPF	FNF	FPF	TNF						
*CFD*	0.982	478	24	1	2	42	<1×10^−10^	0.96	0.95	97.7	92.3	0.91
*EDN3*	0.968	42	23	2	4	40	<1×10^−10^	0.92	0.91	95.2	85.2	0.83
*WISP2*	0.926	151	24	1	10	34	2.1×10^−8^	0.96	0.77	97.1	70.6	0.73

AUC: area under the curve, FPF: false positive fraction, TNF: true negative fraction, TPF: true positive fraction, FNF: false negative fraction, PPV: Positive predictive value, NPV: Negative predictive value.

a Optimal cut-off values (ng/ml) were selected according to the ROC analysis.

b Chi square test.

c J = sensitivity+specificity − 1.

### Analysis of *CCNB2*, *CDC20*, *PRC1*, *SYCP2*, *NUSAP1*, and *CDKN3* Expression in Pre-invasive Neoplasias

For screening tests, it is important to detect not only CC, but also high-grade lesions (CIN2/3) and to distinguish them from low-grade CIN lesions (CIN1) and healthy controls. Therefore, to investigate whether these genes can differentiate CIN2+ from CIN1-, expression was analyzed in 2 additional groups of samples: 29 low-grade CINs and 21 high-grade CINs. Their expression was compared with that of 3 known markers associated with CC (*PCNA*, *MKI67*, and *CDKN2A*), which were ranked in the top 10 in the previous qRT-PCR analysis (see above). Experimental data were box plotted (Figure 4) and the statistical significance of differences was calculated using the MW test. According to the median and distribution of the data in the box plots, the 9 markers can be classified into 3 groups; the first group included markers linked exclusively (*CCNB2*, *PRC1*) or mostly (*SYCP2*) to invasion, which clearly differentiated invading tumors from high-grade CIN, low-grade CIN, and normal cervices. The expression of these markers in the control group, and in low-grade and high-grade lesions was similar (p>0.05, MW). In contrast, the difference between the CC and control samples was quite large, as was established in the previous analysis (Table 2). Similarly, the FC compared to high-grade CIN was also very high, especially for *SYCP2* (FC = 84.8; p<1×10^−15^), followed by *PRC1* (FC = 39.4; p<1×10^−15^, MW) and *CCNB2* (FC = 15.9; p<1×10^−15^ MW). Moreover, the specificity for detecting just CC, and not other lesions, ranged from 0.85 (*SYCP2*) to 0.98 (*CCNB2*); the optimal cut-off values were at a change well over 4.5 fold. The lower specificity shown by *SYCP2* was because 7 preinvasive lesions (5 CIN2/3 and 2 CIN1) had a FC greater than the optimal cut-off value for this gene (7.9). The second group included 4 genes (*CDC20*, *NUSAP1*, *CDKN2A*, and *CDKN3*) the expression of which tended to increase from the control group to the CC group (*CDC20*, *CDKN2A*, and *CDKN3*) or the high-grade CIN group (*NUSAP1*). For *NUSAP1*, the expression in CIN2/3 and CC was similar (Figure 4). These 4 genes could distinguish CIN2+ lesions from CIN1− lesions (p<1×10^−15^, MW; Figure 4). The third group included *MKI67* and *PCNA*, the expression of which increased from the control group to the low-grade CIN group (p<0.05, MW), was similar in the low-grade and high-grade CIN groups (p>0.05, MW), and then increased in the CC group (p<1×10^−15^, MW; Figure 4). It is clear that genes in the first and third groups would not be good markers for screening since they cannot distinguish high-grade CIN and CC lesions from low-grade CIN lesions and control samples. ROC analysis was performed to explore the potential of the genes in the second group (*CDC20*, *CDKN2A*, *CDKN3*, and *NUSAP1*) as markers for screening. None of them had AUC values equal to or greater than 0.97; the highest AUC value was obtained with *CDKN2A* (0.92), followed by *NUSAP1* (0.917), *CDKN3* (0.91) and *CDC20* (0.86) (Table 4). However, the new markers (*NUSAP1* and *CDKN3*) showed a slightly greater sensitivity than *CDKN2A*, while the opposite was true for the specificity (Table 4). Interestingly, the sensitivity and specificity increased when individual data for *CDKN3*, *NUSAP1*, and *CDKN2A* were combined (Table 4). This combination showed the highest Jouden index. From these, only *CDKN3* can also discriminate CC from CIN2/3 (FC cut-off = 4.4) with high sensitivity (0.9) and specificity (0.84).

**Table 4:** ROC analysis of 4 gene markers selected for detection of CIN2/3 and CC.

			≤CIN1 (n = 54)^a^		≥CIN2/3 (n = 65)^a^						
Marker	AUC	Cut-off value	FPF	TNF	TPF	FNF	Sensitivity	Specificity	PPV	NPV	Youden Index
*CDKN2A*	0.920	14	4	50	52	13	0.80	0.93	92.9	79.4	0.73
*NUSAP1*	0.917	71	6	48	59	6	0.91	0.89	90.8	88.9	0.80
*CDKN3*	0.909	50	8	46	55	10	0.85	0.85	87.3	82.1	0.70
*CDC20*	0.854	11	7	47	46	19	0.71	0.87	86.8	71.2	0.58
*CDKN3, CDKN2A, CDC20*			4	50	55	10	0.85	0.93	93.2	83.3	0.77
*CDKN3, CDKN2A, NUSAP1*			4	50	57	8	0.88	0.93	93.4	86.2	0.80
*CDKN3, CDC20, NUSAP1*			6	48	57	8	0.88	0.89	90.5	85.7	0.77
*CDKN2A, CDC20, NUSAP1*			4	50	53	12	0.82	0.93	93.0	80.6	0.74

See legends of Table 3.

The last 4 rows included the combined analysis of CDKN3, NUSAP1, CDC20 and CDKN2A as indicated. Samples were considered positive when at least 2 of the 3 markers were positive.

a All comparisons gave a p-value <1×10^−9^, chi square.

### Verification of the Protein Expression of Selected Tumor Marker Candidates by Immunohistochemistry

**Figure 5:**
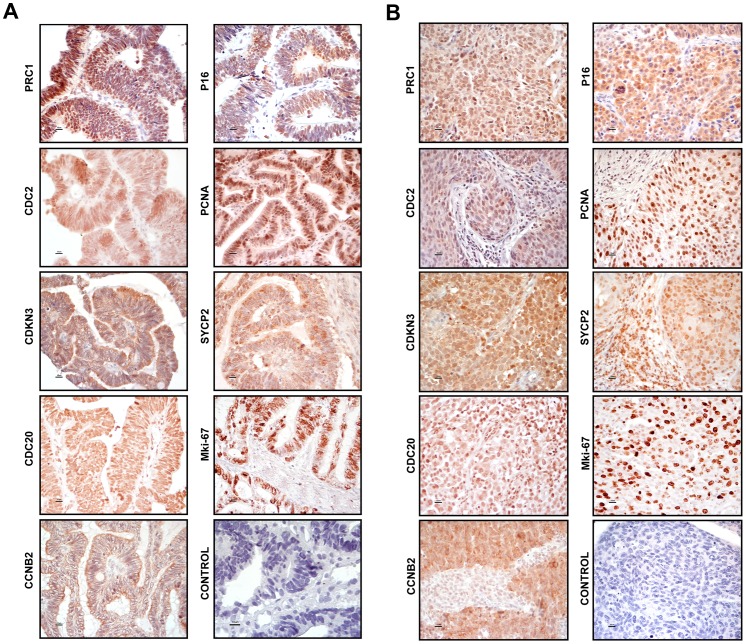
Histological analysis of marker genes. Protein expression was determined by immunohistochemistry using sections from formalin-fixed, paraffin-embedded tissue. Proteins explored were CDKN3A, SYCP2, PRC1, CDC20, CCNB2, PCNA, CDKN2A, MKI67, and CDC2. Representative experiments in adeno cell carcinomas (panel A) and squamous cell carcinomas (panel B) are shown. The specific signals are shown as brown staining (counterstained with hematoxylin; original magnification, ×400; bars, 10 µm).

**Figure 6:**
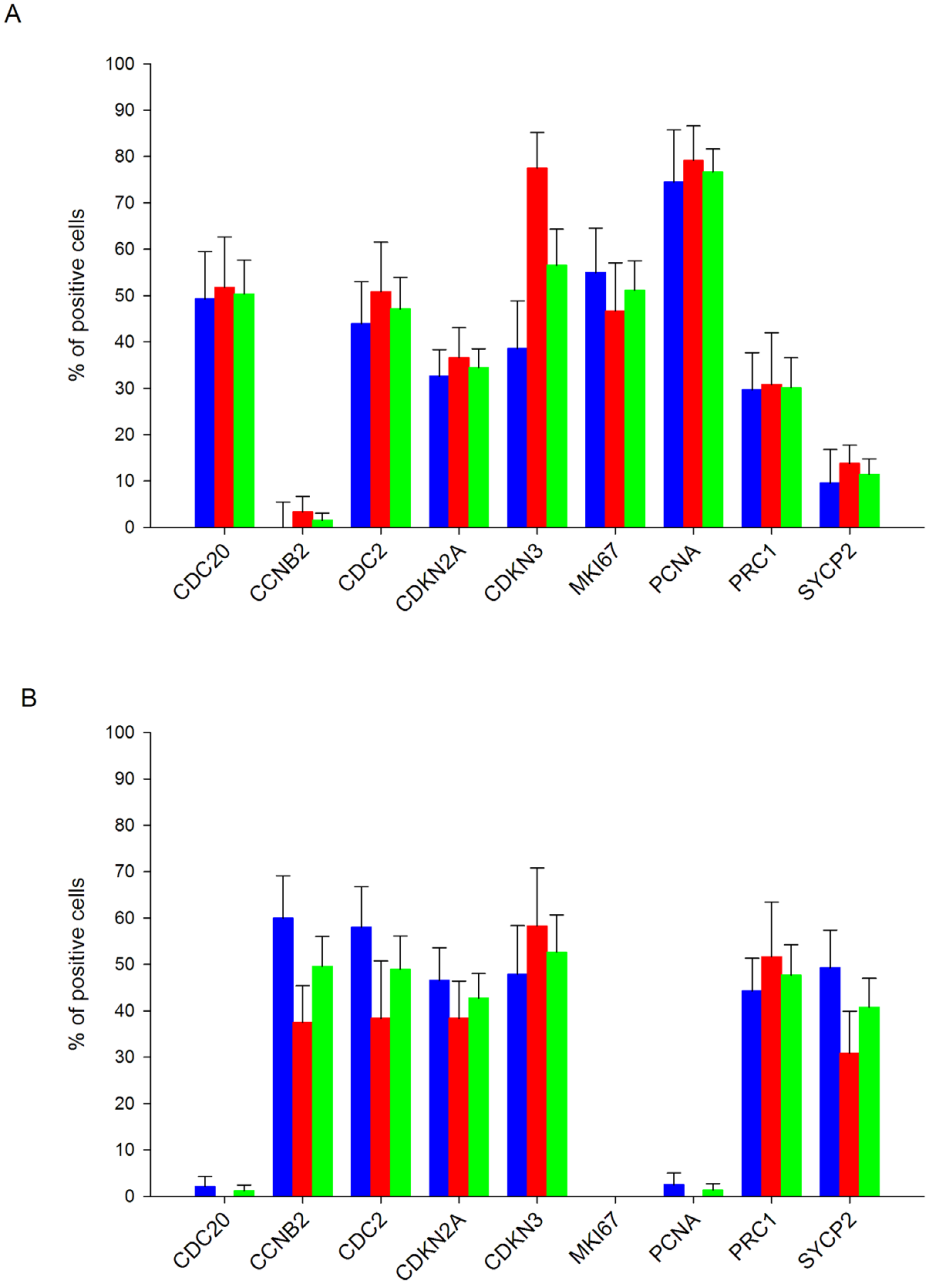
Percentage of tumor cells with positive signals for the markers tested by immunohistochemistry. The average percentages of tumor cells with positive signals in the nucleus (panel A) or cytoplasm (panel B) for CDC20, CCNB2, CDC2, CDKN2A, CDKN3, MKI67, PCNA, PRC1, and SYCP2 are plotted. Tumors positive for HPV16 (blue bars) are compared with tumors positive for other HPVs (red bars). As NUSAP1 was only explored in HPV16-positive CCs, the average percentages of tumor cells with positive signals in nuclei (32.5%) and cytoplasm (68.4%) were not included in this figure. The numbers for the entire set of tumors is also included (green bars). The standard error is shown.

To investigate whether the validated genes (*PRC1*, *CDKN3*, *CCNB2*, *SYCP2*, *NUSAP1* and *CDC20*) were also overexpressed at the protein level, the coding proteins were assessed by IH. The expression of *PCNA*, *CDKN2A*, *MKI67*, and *CDC2* was also examined. All but one (NUSAP1) proteins were explored in 36 samples (10 controls and 26 CCs, 14 positive for HPV16 and 12 positive for other HPVs). NUSAP1 was explored only in HPV16-positive CCs and 5 controls. Unlike the controls, almost all CCs were positive for the 10 antigens (Figure 5, Figure S3). A higher percentage of positive tumors and more intense signals were observed for PCNA (96.2%), followed by CDKN2A and CDKN3 (84.6%), CCNB2 and CDC2 (80.8%), NUSAP1 (79%), MKI67, SYCP2 and PRC1 (76.9%), and CDC20 (73.1%). Unexpectedly, a considerable number of controls were positive for CDC20 (60%), NUSAP1 (40%) and SYCP2 (50%); however, for CDC20 the signals were only observed in the nuclei of cells in the basal layer, for NUSAP1 the signals were observed in the nuclei and cytoplasm of cells in the basal and parabasal layers and for SYCP2 in the basal pole of epithelial cells of superficial and intermediate layers. For the rest of antigens, the differences in positivity between the 2 groups agreed with the data obtained with qRT-PCR (Table S4). Signals for CDKN3, SYCP2, PRC1, CDC2, NUSAP1, and CDKN2A were observed in both the cytoplasm and the nucleus, while signals for CCNB2 were only observed in the cytoplasm, and signals for CDC20, PCNA, and MKI67 were only observed in the nucleus (Figure 5, Figure S3). As expected, the IH signals were not uniform in all cells of all tissues, but rather the distribution was heterogeneous, indicating that not all cells are at the same stage of the cell cycle. The PCNA signals showed the most uniform distribution, and on average 70% of the nuclei were positive, suggesting that approximately 70% of the cells in the tissues were in S phase of the cell cycle. For the rest of the proteins, nuclear signals were observed in 10–50% of cells (Figure 6A). Signals for the proteins localized in the cytoplasm were observed in 40–50% of cells on average (Figure 6B). Given that all these proteins are involved in the M phase of the cell cycle (see below and discussion), the data suggest that 30–40% of the cells are in some stage of this phase. Interestingly, the percentage of cells positive for CCNB2, CDC2, and SYCP2 was higher in tumors positive for HPV16 than in tumors positive for other HPVs, and the opposite was observed for CDKN3 (Figure 6). The predictive capability of IH was also evaluated. Compared to the RT-PCR results, the sensitivity was lower for all proteins, but the specificity was higher for all proteins, except for SYCP2, NUSAP1 and CDC20 (Table S4).

### Molecular Targets in Cervical Cancer Associated with Poor Survival

**Figure 7:**
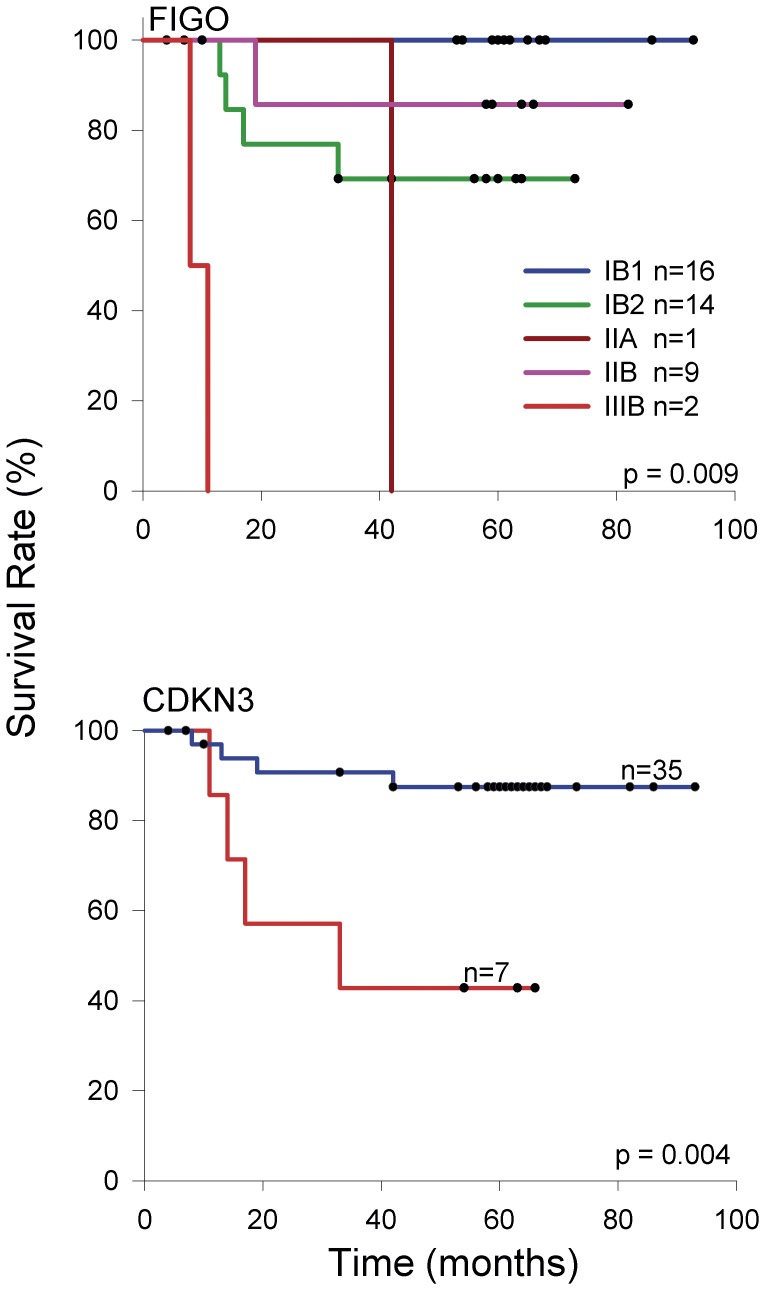
Survival analysis of women with cervical cancer according to FIGO staging and *CDKN3* expression. The Kaplan-Meier curves for FIGO staging and *CDKN3* are shown. Patients were followed-up for at least 42 months. For gene expression, cancer patients with higher (red line) and lower (blue line) fold change values were compared (see material and methods). The p value was calculated by comparing the curves with the log-rank test. Censored patients are labeled with black dots, but only four of them were censored before the minimal period of follow-up (42 months).

One way to investigate whether or not these molecular targets are associated with cervical cancer progression is a survival study. Therefore, a survival analysis using the qRT-PCR expression values of *PRC1*, *CCNB2*, *CDC20*, *CDKN3*, *NUSAP1*, SYCP2, *CDKN2A*, *PCNA*, and *MKI67* and FIGO staging was conducted on 42 patients with HPV16-positive CC whose progress was followed-up for at least 3.5 years after their diagnosis and initial treatment (Table 1). This subset included FIGO stages IB1 (n = 16), IB2 (n = 14), IIA (n = 1), IIB (n = 9), and IIIB (n = 2). The overall survival rate for the whole sample was 79.6% and for FIGO stages IB1, IB2, IIA, IIB, and IIIB were 100%, 69.2%, 0%, 85.7%, and 0%, respectively. These differences were statistically significant (p<0.001, log-rank test; Figure 7A). Of the 9 genes analyzed using Kaplan-Meier curves, only *CDKN3* was associated with poor survival (p = 0.004, log-rank test; Figure 7B). The overall survival rate of patients with the higher levels of *CDKN3* (FC >15) was 42.9%, and the median survival time was 33 months. In contrast, those with lower levels of *CDKN3* had an overall survival rate of 87.5%. FIGO staging and *CDKN3* expression were analyzed individually and together in Cox proportional hazard models. Because of the differences in the sample size among the FIGO stages analyzed, patients were reassigned to 2 groups, one including FIGO IB1 and IB2 (n = 30) and the other FIGO IIA, IIB, and IIIB (n = 12). Individually, the hazard ratio (HR) of *CDKN3* was 5.9 (95% CI 1.4–24.1, p = 0.01) and of the grouped FIGO, 3.3 (95% CI 0.83–13.3, p = 0.08). The lack of significance in the HR of grouped FIGO could be explained by differences in the sample size and the inverted survival rates of the individual FIGO stages IB2 and IIB. When these 2 covariates were included in the same proportional hazard model, *CDKN3* remained invariably significant with an HR of 5.9 (95% CI 1.4–23.8, p = 0.01). These results suggest that *CDKN3* could be a prognostic factor for survival that is independent of FIGO staging. However, a larger sample size is needed to confirm these results.

### Classification of Genes with Differential Expression between Cancer and Control Samples

**Figure 8:**
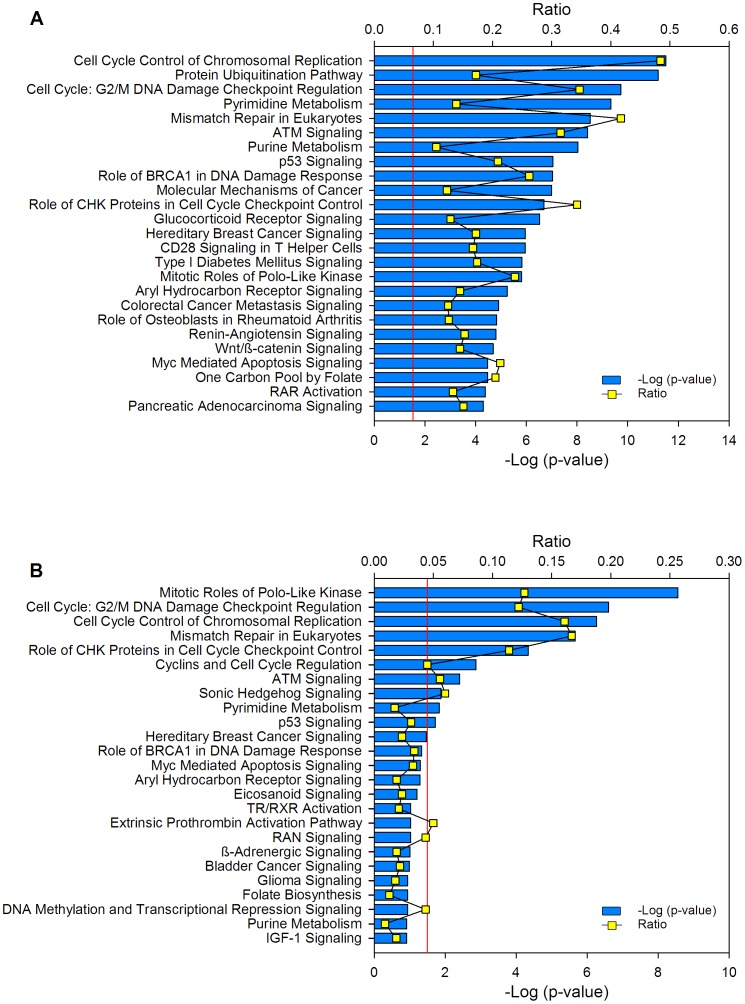
Canonical pathways where deregulated genes are involved. Top 25 canonical pathways identified in the set of 997 deregulated genes between the tumors and controls (A) and in the subset of the 100 best-ranked genes (50 upregulated and 50 downregulated; B). The canonical pathways were identified using the Ingenuity Pathway Analysis (IPA) system. The −log (p-value) (blue bars) and the ratio (yellow dots) were calculated by comparing the number of genes in the pathways present in the datasets versus the human database. The p-value was calculated using the chi square test or Fisher’s exact test as appropriate, and the -log (p-value) values >1.3 (red line) correspond to *p*<0.05.

The DAVID functional annotation tool (http://david.abcc.ncifcrf.gov) was used at medium and highest stringency to identify the biological processes where the 997 differentially expressed genes are involved. Compared with the human genome database, the 3 most enriched clusters, and with the lowest p values at medium stringency, were cell cycle-associated processes, DNA metabolic processes, and processes associated with the regulation of ubiquitin-protein ligase activity (Table S5). Interestingly, at the highest stringency, where more tightly associated genes in each group are expected, the clusters including mitosis and M-phase of mitotic cell-cycle processes were ranked in the 1st, 2nd, and 5th places (Table 5). It is worth noting that, among the cell cycle processes, none except the M-phase was enriched significantly (Table 5). Remarkably, in the 100 top ranked genes subset (50 upregulated and 50 downregulated), the mitosis cluster was also the most enriched pathway and compared with the whole set (n = 997) it was enriched over 3.3 fold (Table 6). This data indicated that the genes involved in mitosis were not only the most enriched, but also the most different in terms of FC and Δ-score, when compared with the control samples (circles in red and orange; Figure 1). In fact, 11 of the 21genes associated with CC and validated in this work (*CCNB2*, *CDC20*, *PRC1*, *SYCP2*, *NUSAP1*, *CDKN3, CDC2, CKS2, MKI67, SMC4* and *ZWINT*) are involved in the M phase of the cell cycle. The data were also analyzed with the IPA Ingenuity system and the findings were similar to those obtained with DAVID, especially when the DAVID analysis was run with medium stringency (Table S5). In agreement with the DAVID analysis, the protein ubiquitination pathway was the second top canonical pathway in the entire set of deregulated genes (Figure 8A) and the mitotic roles of polo-like kinase was the top in the subset of the 100 top ranked genes (Figure 8B).

**Table 5:** DAVID functional annotation cluster analysis at the highest stringency of 997 genes deregulated in cervical cancer*.

Cluster	Enrichment Score	Biological Process	No. Genes	p-value	Fold Change
1	16.24	Mitosis	54	3.00E-17	3.8
		nuclear división	54	3.00E-17	3.8
		M phase of mitotic cell cycle	54	6.80E-17	3.7
		organelle fission	54	1.90E-16	3.6
2	14.02	positive regulation of ubiquitin-protein ligase activity duringmitotic cell cycle	28	3.60E-15	6.3
		positive regulation of ligase activity	28	2.90E-14	5.9
3	5.71	RNA splicing, via transesterification reactions	28	1.90E-06	2.8
		nuclear mRNA splicing, via spliceosome	28	1.90E-06	2.8
4	3.66	positive regulation of apoptosis	49	1.90E-04	1.7
5	3.03	mitotic cell cycle spindle assembly checkpoint	6	3.90E-04	8.3
		regulation of mitotic metaphase/anaphase transition	6	1.20E-02	4.2
6	2.81	mesenchymal cell differentiation	11	1.50E-03	3.3
7	2.34	somatic cell DNA recombination	7	3.70E-03	4.5
		somatic diversification of immune receptors	7	6.90E-03	4.0
8	2.01	carboxylic acid metabolic process	52	8.70E-03	1.4
		cellular ketone metabolic process	52	1.20E-02	1.4
9	1.96	establishment of RNA localization	14	1.00E-02	2.2
		RNA transport	14	1.00E-02	2.2
10	1.82	Glycolysis	10	3.00E-03	3.3
		hexose catabolic process	10	3.50E-02	2.2
11	1.73	embryonic epithelial tube formation	8	2.00E-02	2.8
12	1.73	somatic recombination of immunoglobulin gene segments	6	4.90E-03	5.1
		production of molecular mediator of immune response	6	4.90E-02	3.0
13	1.56	provirus integration	4	1.20E-02	7.6
		DNA integration	4	1.40E-01	3.1
14	1.54	natural killer cell mediated cytotoxicity	4	1.20E-02	7.6
		leukocyte mediated cytotoxicity	4	2.40E-02	6.1
15	1.39	regulation of caspase activity	11	3.20E-02	2.1
		regulation of peptidase activity	11	5.30E-02	2.0
16	1.38	somatic recombination of immunoglobulin genes during immune response	4	3.90E-02	5.1
		immunoglobulin production during immune response	4	4.90E-02	4.7

* Enrichment Score is the -log_10_ of the average p-value of the terms in the cluster. Fold change is the ratio of the proportion of genes in the tested list versus the Human Gene Reference database.

**Table 6:** DAVID functional annotation cluster analysis at the highest stringency of the 100 genes most deregulated in cervical cancer compared with normal cervical epithelium*.

Cluster	EnrichmentScore	Biological Process	No. Genes	p-value	FoldChange
1	13.89	Mitosis	19	1.00E-14	12.0
		nuclear división	19	1.00E-14	12.0
		M phase of mitotic cell cycle	19	1.40E-14	12.0
		organelle fission	19	2.00E-14	12.0
2	3.49	regulation of ubiquitin-protein ligase activity	6	2.10E-04	11.0
3	2.87	negative regulation of ubiquitin-protein ligase activity	5	1.20E-03	11.0
4	2.83	positive regulation of mitosis	4	6.70E-04	23.0
		positive regulation of cell cycle	4	7.20E-03	10.0
5	2.75	establishment of mitotic spindle localization	3	1.30E-03	53.0
6	2.58	anaphase-promoting complex-dependent proteasomalubiquitin-dependent protein catabolic process	5	1.10E-03	11.0
		regulation of ubiquitin-protein ligase activity during mitotic cell cycle	5	1.50E-03	10.0
7	2.31	negative regulation of protein modification process	6	1.40E-03	7.2
		negative regulation of protein metabolic process	6	9.70E-03	4.6
8	1.58	regulation of protein modification process	8	4.40E-03	3.9
		regulation of cellular protein metabolic process	8	4.70E-02	2.4
9	1.53	nucleosome assembly	4	2.10E-02	6.8
		chromatin assembly	4	2.30E-02	6.6
10	1.42	regulation of protein kinase activity	7	3.30E-02	2.9
11	1.31	regulation of phosphorylation	8	4.30E-02	2.4

* Enrichment Score is the -log_10_ of the average p-value of the terms in the cluster. Fold change is the ratio of the proportion of genes in the tested list versus the Human Gene Reference database.

## Discussion

In this work we identified 6 genes (*PRC1*, *CCNB2*, *SYCP2 CDKN3*, *NUSAP1*, and *CDC20*) associated with invasive cervical cancer that could be used either as markers for diagnosis or as therapeutic targets. While *PRC1*, *CCNB2*, and *SYCP2* were associated mostly with CC, *CDKN3*, *NUSAP1*, and *CDC20* were found to also be associated with high-grade CIN. We recently examined the expression of these genes in 4 cell lines (SiHa, CaSki, HeLa, and Calo) by using the HG-ST1.0 microarray. Although the overexpression was not uniform in the 4 cell lines, in the global analysis all genes except 1 (*SYCP2*) were observed to be upregulated; *SYCP2* was upregulated only in CaSki and SiHa cells [37]. This finding indicates that these markers are correctly predictive of cervical cancer. *CDKN3*, *NUSAP1*, and *CDC20*, along with *CDKN2A*, can differentiate CC and high-grade CIN from low-grade CIN and normal cervices; therefore, they could be used as markers for screening tests. Furthermore, high expression of *CDKN3* was associated with poor survival of cancer patients; therefore, it also could be used as a survival marker. The sensitivity and specificity of *CDKN3*, *NUSAP1*, and *CDC20* to identify high-grade CIN and CC were as high as 91% and 93%, respectively. Several studies have used microarrays to identify genes associated with cervical cancer [38]–[46]. However, most of them included heterogeneous samples positive for different or undetermined HPV types, a small number of tumors and controls, and essentially their design was not sufficient to identify markers for screening. Therefore, several of the 23 markers validated in this study, in which only HPV16-positive CCs were analyzed with MA, either have not been identified previously (*CDKN3, NUSAP1, SMC4, WISP2*) or have rarely been (*CCNB2, CDC20, CKS2, RRM2*) identified in other studies (Table S6). In contrast, over half (Table S6) [40] or 40% (Table S6) [43] of them have been found in individual studies, and many of them have been identified in 3 or more studies (*CDKN2A, MCM2, PCNA, RFC4, SYCP2*, and *TYMS*) [46]. The fact that *CDKN3, CDC20* and *NUSAP1* were demonstrated upregulated in CIN2+ positive for other or undetermined HPV types by RT-PCR, suggests these genes could be considered potential markers for CC screening regardless of viral type.

Markers for screening that have high sensitivity and specificity have not been identified or reported. In addition to the conventional Pap and Liquid Base Cytology (LBC), different tests for HPV detection are used for screening. Hybrid Capture 2 Technology (HC2) is the methodology most frequently used for screening, particularly for measuring high-risk virus. This method, approved by the FDA in United States, has an average sensitivity of 95% (range, 62–98) for detecting high-grade lesions and invasive cancer. However, this methodology has low specificity, especially in young women, since the majority of infections are not associated with neoplastic lesions [15], [47], [48]. In women over the age of 30 years, the specificity is much higher; however, it is quite variable among studies, and depends in part on the prevalence of HPV in the study population [49]. Furthermore, in most studies, the PPV is very low, less than 30%, which indicates that only that percentage of infected women have high-grade lesions.

It is important to emphasize that the primary value of cervical cancer biomarkers and the goal of developing procedures for cervical screening is to improve the specificity rather than sensitivity relative to HPV testing [11]. Primary HPV DNA screening with cytology triage increases the specificity similar to that of conventional cytology [9], [15]. However, use of this procedure in developing countries creates logistical problems, either because a high percentage of women who test positive for HPV do not return for cytology or due to the handling of samples when taking a sample for cytology from all patients at the first visit. In addition, because it prevents automatization, it seems impractical. The simultaneous use of HC2 for high-risk viruses with a molecular method that distinguishes CIN2+ from CIN1− would increase the specificity and the PPV, with the advantages of being faster and having the potential to be automated compared to triaged cytology.

Of the markers associated with CC, p16, a tumor suppressor protein, is the most studied [11]. This protein accumulates in the nucleus and cytoplasm of cells transformed by high-risk HPVs and is usually detected by IH. The amount of p16 is related to the severity of cervical neoplasia and is considered a marker of CIN2+. P16 has been successfully deployed for the classification of HPV-related disease. For cervical tissue punch and cone biopsies, IH for p16 has been reported to reduce interobserver disagreement when compared with diagnosis of H&E stained sections. P16 has also recently emerged as a sensitive and specific diagnostic adjunct for underlying CIN2+ lesions in cervical cytology specimens [11]. It consistently exhibits high sensitivity (80–95%) for detection of CIN2+; however, the specificity is lower than that for cytology (∼50%) [50], [51]. This is because approximately 38% of low-grade CIN lesions, those infected with high-risk HPV types, express this marker [50]. The relatively low specificity of this marker and the need for a pathologist to interpret the IH are the main reasons why this marker has not been adopted for primary screening. Recently, Wentzensen *et al*., developed methods to detect p16 protein in cell lysates of cervix exudates using ELISA. The sensitivity of this ELISA method for the identification of high-risk lesions was 84%, and the specificity was 87% [52]. In agreement with these data, the specificity of *CDKN2A* mRNA detection, which encodes p16, in screening for CIN2+ was very close (93%, Table 4).

Two new markers identified in this work (*CDKN3* and *NUSAP1*), along with *CDKN2A*, showed a high specificity (93%) and PPV (93.4%); therefore, they might be good candidates to use with HC2 as a first-line strategy in a screening program. The scope of this study was to perform a feasibility evaluation to ascertain whether determining the mRNA levels of novel genes in cervical samples would allow for the identification of high-grade CIN or invasive lesions with high sensitivity and specificity. However, the potential sensitivities reported in this analysis are most likely overestimated compared to those likely to be found in clinical practice, as those with CIN2+ have a higher proportion of cervical cancer (which is easy to identify) than that expected in any screening setting. In contrast, the specificity seems to be underestimated, given that a large proportion of CIN1- had CIN1. Therefore, we did not expect to obtain conclusive data on the sensitivity, specificity or predictive values of the assays. Further studies are needed to determine the levels of *CDKN3*, *NUSAP1*, and *CDC20* mRNA or protein in cervical samples from a screening population to obtain information about the predictive values and to define the optimal trade-off between sensitivity and specificity for the detection of CIN2+.

*PRC1*, *CCNB2*, and *SYCP2* are markers exclusively associated with invasive cervical cancer. Together with *NUSAP1*, *CDKN3*, and *CDC20*, these genes represent potential specific targets for the treatment of advanced CC, particularly *CDKN3*, which was found to be associated with poor survival. These genes encode proteins involved in the cell cycle, specifically in the M phase (mitosis and cytokinesis). According to the IH data, approximately 30% of tumor cells in CC could be in the M phase. These genes participate in anaphase control, chromosome segregation, and mitotic entrance/exit. While activation of cyclin dependent kinases (Cdks) drives cells into mitosis, mitotic exit depends on inhibition of Cdks activity, mainly through degradation of mitotic cyclins by the anaphase-promoting complex (APC/C) and accumulation of Cdk inhibitor proteins, and dephosphorilation of proteins phosphorylated by CDKs. Four (CCNB2, CDC20, CDKN3, PRC1) of the six proteins validated in this paper seem essential in this process. Cyclin B2 (CCNB2), like cyclin B1 (CCNB1), binds to CDK1 (CDC2) to form the complex M-CDK, which is essential for control of the cell cycle at the G2/M transition. However, while cyclin B1-CDK1 causes chromosome condensation, reorganizes microtubules, and disassembles the nuclear lamina and the Golgi apparatus, cyclin B2-CDK1 is restricted to the cytoplasm and disassembles the Golgi apparatus during mitosis [53], [54]. In agreement with these data, cyclin B2 was localized exclusively in the cytoplasm of the CCs examined in this paper (Figure 5). Interestingly, the expression of cyclin B1 in these tumors did not differ from that in the control samples (Table S3). This cyclin is degraded by the APC/C, a key regulator of the metaphase-to-anaphase transition, to allow progression of mitosis from metaphase to anaphase [55]. *CCNB2* has been scantly associated with cervical cancer [40]; however, it has been reported to be associated with other types of cancer. For instance, it is upregulated in cancers of the colon [56], lung, and digestive tract [57]. The increased amount of CDC20, a key regulatory protein of APC/C complex during anaphase, could explain the absence of cyclin B1. CDC20, together with UBE2C (also known as UBCH10), which was also increased in CC (Table S3), is required for full ubiquitin ligase activity of the APC/C complex and may confer substrate specificity upon the complex. CDC20 is negatively regulated by MAD2L1 and BUB1B (also known as BUBR1). In metaphase the MAD2L1-CDC20-APC/C ternary complex is inactive, while in anaphase the CDC20-APC/C binary complex is active in degrading substrates. Interestingly, the MAD2L1 and BUB1B transcripts were also increased in CC (Table S3) suggesting that the corresponding proteins could be increased and prevent activation of APC/C. However, part of the CDC20 protein could remain free to bind and activate APC/C, as has been shown in transfected cells expressing the E6/E7 proteins [55]. CDC20 has been found to be upregulated in lung, pancreatic, and gastric cancers [58], as well as in CC [40], [59]. CDKN3 is a dual-specificity protein phosphatase of the Cdc14 phosphatase group that interacts with CDK1 (CDC2) and inhibits their activity [60], [61]. CDKN3 and other Cdc14 phosphatases have not been well studied; however, they seem to be essential for antagonizing Cdk activity in late mitosis, allowing cells to exit mitosis in telophase. Regulation of cytokinesis may be the 1 conserved function of the Cdc14 phosphatases. Although overexpression of CDKN3 has been associated with inhibition of cell proliferation in colon cancer cell lines [62], it has also been found to be overexpressed in breast, prostate, and lung cancers [63]–[65]. In agreement with our data, *CDKN3*, along with other genes, has been found to be associated with lower survival of patients with lung adenocarcinomas [63]. This is the first report in which *CDKN3* was associated with cervical cancer (Table S6). PRC1 is involved in cytokinesis and is essential for controlling the spatiotemporal formation of the midzone and successful cytokinesis [66], [67]. It is required for kinesin-family member 14 (KIF14) [68] and polo-like kinase 1 (PLK1) [69] localization to the central spindle and midbody. The suppression of PRC1 blocks cell division. The transcription of *PRC1* is repressed by p53 and is one of the routes by which p53 stops the cell cycle at the G2/M checkpoint [70]. Since the E6 oncoprotein of HPV16 induces degradation of p53 in proteasomes, it is likely that in cervical carcinomas PRC1 is being overexpressed via this mechanism. It has been reported to be associated with liver cancer [71] and CC [40], [42]. NUSAP1 is a nucleolar-spindle-associated protein that plays a role in spindle microtubule organization. This gene has not been described as associated with CC, but has been found to be upregulated in breast and melanoma cancers [72]. SYCP2 is a major component of the synaptonemal complex. This complex promotes that double strand breaks (DSB) are repaired by the homologous recombination pathway in meiosis [73]. The high levels of *SYCP2* expression in the CCs examined in this work suggests that DSB are very common in some CC samples and that SYCP2 could be involved in DSB repair by the stimulation of homologous recombination pathway. Interestingly, this gene has been found to be upregulated in CC [45], [46] and oropharyngeal squamous cell carcinomas positive for HPV16, but not in HPV-negative carcinomas [74].

Cell cycle is the main process altered in CC and is top ranked in all CC papers where biological processes have been analyzed [46]. Similarly, in the present paper, when the gene dataset was analyzed using the DAVID tool at medium stringency, the cell cycle process was shown to be the most enriched and it ranked at the top of the list (Table S5). However, the fact that M-phase processes were the most enriched in our dataset when the analysis was done at high stringency, suggests that the M-phase is the main altered cell-cycle phase in CC. These findings are consistent with the alterations in the cell cycle and mitosis caused by HPV *in vitro* [59], [75], [76] and correlated in few CC studied [59]. The E6 and E7 oncoproteins of high-risk HPVs induce numerous mitotic defects, including multipolar mitoses, chromosomal missegregation, anaphase bridges, and aneuploidy. Although cells with abnormal mitoses are normally targeted for cell death, E6 and E7 act cooperatively to allow cells with abnormal centrosomes to accumulate by relaxing the G2/M checkpoint response and inhibition of apoptotic signaling [76]. In agreement with these data, the canonical pathways of G2/M DNA Damage Checkpoint Regulation and the Role of CHK Proteins in Cell Cycle Checkpoint Control ranked at the second and fifth positions of the altered canonical pathways in CC. On the other hand, E6 and E7 induce mechanisms to avoid mitosis checkpoint. The E6/E7 genes have been shown to induce the overexpression of CDC20 and UBCH10, which activate the APC/C ubiquitin ligase complex [55]. The enrichment of positive regulation of ubiquitin-protein ligase activity during mitotic cell cycle found in CCs (Table 5) completely agree with these *in vitro* results.

Inhibition of mitosis is a well-known strategy to combat cancers. Drugs that perturb the process of cell division have proved to be effective anticancer therapies. Well-known examples of these drugs are those that perturb the formation of the mitotic spindle, such as taxanes and vinca alkaloids. However, they have remarkably low therapeutic indices and narrow therapeutic windows. Their efficacy is restricted because they also perturb the microtubule network of non-dividing cells, causing neurotoxic effects and affecting endothelial cell function. To resolve this issue, a new generation of antimitotic agents has been developed that target kinesins and kinases with unique roles in mitosis, such as KIF11, PLK1, and aurora kinase A (AURKA) [69]. Interestingly, the transcripts of these 3 genes were also upregulated in the CCs (Table S3), *AURKA* ranked in 19th place, *KIF11* ranked in 72nd place, and *PLK1* ranked in 263rd place. Therefore, those drugs could be tested for the treatment of cervical cancer. On the other hand, the high FC of the novel genes validated in this work, especially *CDKN3*, *CDC20*, and *SYCP2*, compared with the control samples, makes these genes potential targets for CC therapy. However, it is still necessary to demonstrate whether they are indispensable for tumor growth.

## Supporting Information

Figure S1 **Correlation of expression intensity of 997 genes examined by HG-Focus and HG-ST1.0 microarrays.** The Log_2_ values of the standardized intensity signals (RMA values) of 997 genes in a typical tumor (R230) examined by the 2 microarrays were plotted. The linear trend (black line) is included, which was calculated with Person’s correlation test: r = 0.78, p<1×10^−15^.
(TIF)

Figure S2 **Validation of gene expression of 3 genetic markers by qRT-PCR.** The intensity of gene expression, expressed in Log_2_ values, is shown in box plots. Expression of the 3 novel downregulated genes revealed in this study (CFD, EDN3, WISP2) associated with CC are compared between healthy cervical epitheliums (Control, n = 25) and invasive CC (Tumor, n = 44). See legend of Figure 4.
(TIF)

Figure S3 **Histological analysis of NUSAP1.** Protein expression was determined by immunohistochemistry using sections from formalin-fixed, paraffin-embedded tissue. Representative experiments in adeno cell carcinomas (left panel) and squamous cell carcinomas (right panel) are shown. The specific signals are shown as brown staining (counterstained with hematoxylin; original magnification, ×400; bars, 10 µm).
(TIF)

Table S1 **Summary of clinical data, HPV type and methodology to explore gene expression.** SCC, squamous cell carcinoma; ACC, adenocarcinoma; ASCC, Adenosquamous; NCE, normal cervical epithelium; CIN, cervical intraepithelial neoplasia; MA, microarray; MA1, HG-Focus; MA2, HG-ST1.0; qRT-PCR, real-time RT-PCR; IH, Immunohistochemistry; ND, not done. a. Control samples codes ending with X are exocervix and ending with E are endocervix.
(XLSX)

Table S2 **TaqMan gene expression assays from Applied Biosystems.**
(XLSX)

Table S3 **List of the 997 genes differentially expressed in cervical carcinomas compared with normal cervical epitheliums.** a. Genes were ranked by Δ-score and those marked in bold were selected for analysis with real time RT-PCR. b. The tumor/control fold change (FC) of each gene was calculated using the mean values of signal intensity obtained with the SAM method. c. Genes which have been used as biomarkers in different diseases, according with the IPA Ingenuity system: D, Diagnosis; DP, Disease Progression; P, Prognosis; E, Efficacy; RT, Response to Therapy; UP, Unspecified Application; S, Safety.
(XLSX)

Table S4 **Analysis of 10 proteins in 26 CC and 10 normal cervical epithelium samples by IH and calculus of sensitivity, specificity and predictive values.**
(XLSX)

Table S5 DAVID functional annotation cluster analysis at medium stringency of 997 genes desregulated in cervical cancer*. * Enrichment Score is the -log_10_ of the average p-value of the terms in the cluster. Fold change is the ratio of the proportion of genes in the tested list versus the Human Gene Reference database.
(XLSX)

Table S6 **Comparison of the 23 genes identified and validated in this study with previously reported microarray analysis.** a Cell line immortalized human keratinocyte lines NIKS-16. b. Detected in HeLa cells.
(XLSX)
