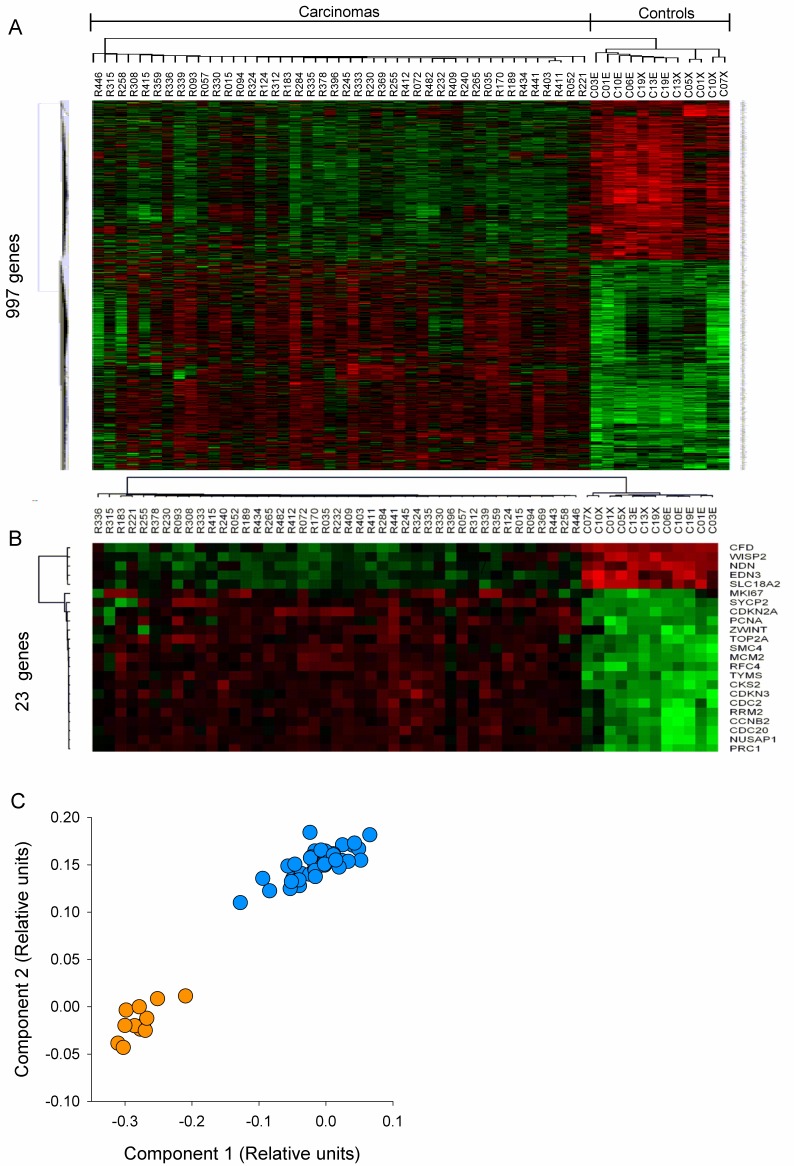# Correction: Mitosis Is a Source of Potential Markers for Screening and Survival and Therapeutic Targets in Cervical Cancer

**DOI:** 10.1371/annotation/36613e7a-41fa-4199-87eb-40214100b4cb

**Published:** 2013-10-30

**Authors:** Ana María Espinosa, Ana Alfaro, Edgar Roman-Basaure, Mariano Guardado-Estrada, Ícela Palma, Cyntia Serralde, Ingrid Medina, Eligia Juárez, Miriam Bermúdez, Edna Márquez, Manuel Borges-Ibáñez, Sergio Muñoz-Cortez, Avissai Alcántara-Vázquez, Patricia Alonso, José Curiel-Valdez, Susana Kofman, Nicolas Villegas, Jaime Berumen

An error was introduced in the preparation of this article for publication. In Table 4, the titles of the third and fourth columns are combined. The correct titles for the third and fourth columns are "Cut-off value" and "FPF" respectively.

In Figure 2A, the label "C07X" is not included on the control samples in the far right column. Please view the correct Figure 2 here: 

**Figure:**