# Supplementary figures and images for: Mitosis Is a Source of Potential Markers for Screening and Survival and Therapeutic Targets in Cervical Cancer
Source: PLoS One. 2013 Feb 6;8(2):e55975. doi: 10.1371/journal.pone.0055975 (PMC3566100; doi:10.1371/journal.pone.0055975)

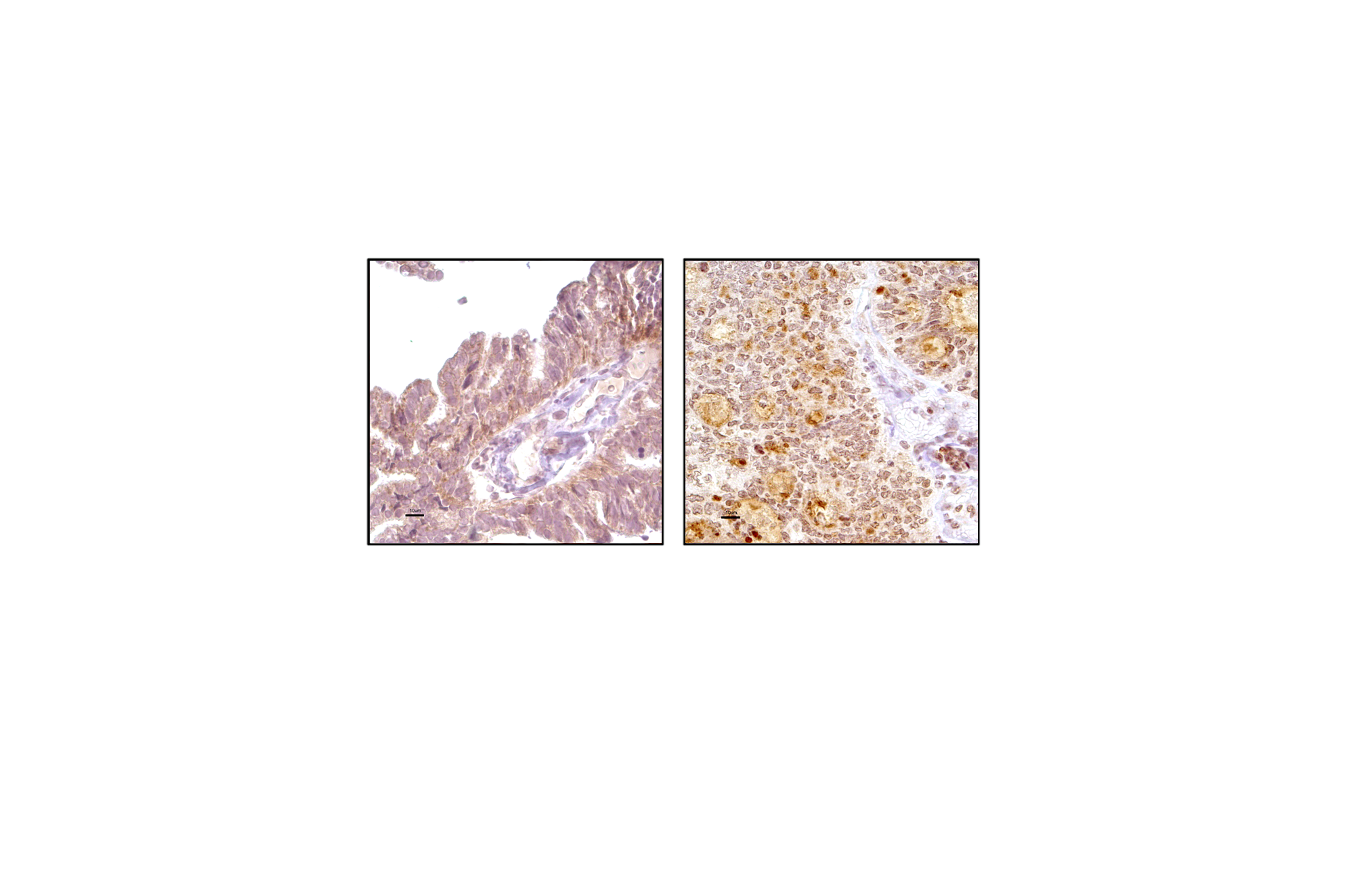

Supplement: Figure S3 — Histological analysis of NUSAP1. Protein expression was determined by immunohistochemistry using sections from formalin-fixed, paraffin-embedded tissue. Representative experiments in adeno cell carcinomas (left panel) and squamous cell carcinomas (right panel) are shown. The specific signals are shown as brown staining (counterstained with hematoxylin; original magnification, ×400; bars, 10 µm). (TIF) [file pone.0055975.s003.tif]
